# Deletion of Pyruvate Carboxylase in Tubular Epithelial Cell Promotes Renal Fibrosis by Regulating SQOR/cGAS/STING‐Mediated Glycolysis

**DOI:** 10.1002/advs.202408753

**Published:** 2025-01-21

**Authors:** Hao Huang, Yuanyuan Han, Yan Zhang, Jianhua Zeng, Xin He, Jiawei Cheng, Songkai Wang, Yiwei Xiong, Hongling Yin, Qiongjing Yuan, Ling Huang, Yanyun Xie, Jie Meng, Lijian Tao, Zhangzhe Peng

**Affiliations:** ^1^ Department of Nephrology, Xiangya Hospital Central South University Changsha 410008 China; ^2^ Department of Cell biology School of Life Sciences Central South University Changsha 410013 China; ^3^ Hunan Key Laboratory of Organ Fibrosis Central South University Changsha 410013 China; ^4^ FuRong Laboratory Changsha 410008 China; ^5^ National Clinical Research Center for Geriatric Disorders Central South University Changsha 410008 China; ^6^ National Medical Metabolomics International Collaborative Research Center Central South University Changsha 410008 China; ^7^ Department of Pathology, Xiangya Hospital Central South University Changsha 410008 China; ^8^ Department of Pulmonary and Critical Care Medicine, Third Xiangya Hospital Central South University Changsha 410013 China

**Keywords:** cGAS‐STING, glycolysis, pyruvate carboxylase, renal fibrosis, SQOR

## Abstract

Renal fibrosis is a common pathway involved in the progression of various chronic kidney diseases to end‐stage renal disease. Recent studies show that mitochondrial injury of renal tubular epithelial cells (RTECs) is a crucial pathological foundation for renal fibrosis. However, the underlying regulatory mechanisms remain unclear. Pyruvate carboxylase (PC) is a catalytic enzyme located within the mitochondria that is intricately linked with mitochondrial damage and metabolism. In the present study, the downregulation of PC in various fibrotic animal and human kidney samples is demonstrated. Renal proximal tubule–specific *Pcx* gene knockout mice (*Pcx^cKO^*) has significant interstitial fibrosis compared to control mice, with heightened expression of extracellular matrix molecules. This is further demonstrated in a stable PC knock‐out RTEC line. Mechanistically, PC deficiency reduces its interaction with sulfide:quinone oxidoreductase (SQOR), increasing the ubiquitination and degradation of SQOR. This leads to mitochondrial morphological and functional disruption, increased mtDNA release, activation of the cGAS‐STING pathway, and elevated glycolysis levels, and ultimately, promotes renal fibrosis. This study investigates the molecular mechanisms through which PC deficiency induces mitochondrial injury and metabolic reprogramming in RTECs. This study provides a novel theoretical foundation and potential therapeutic targets for the pathogenesis and treatment of renal fibrosis.

## Graphical abstract

Pyruvate carboxylase (PC), as a catalytic enzyme located within the mitochondria, plays an important role in kidney fibrosis. PC deficiency reduces its interaction with sulfide:quinone oxidoreductase (SQOR), increasing the ubiquitination and degradation of SQOR. This leads to mitochondrial morphological and functional disruption, increased mtDNA release, activation of the cGAS‐STING pathway, and elevated glycolysis levels; and ultimately, promotes renal fibrosis.

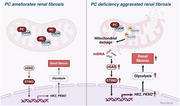

## 1 Introduction

Chronic kidney disease (CKD) is a progressive disease that has various etiologies and causes structural and functional changes in the kidneys.^[^ ^1^ ^]^ Approximately 10% of adults worldwide have CKD, resulting in 1.2 million deaths each year.^[^ ^2^ ^]^ The prevalence of CKD in China is 10.8%, and the number of patients has reached 132.3 million.^[^ ^3^, ^4^ ^]^ In addition, patients with CKD are at an increased risk of acute kidney injury (AKI), and repeated episodes of AKI can aggravate the progression of CKD (AKI to CKD).

Renal fibrosis is an important pathologic basis and common pathway for the progression of various CKDs to end‐stage renal disease; furthermore, it is the main determinant of gradual loss of renal function.^[^ ^5^ ^]^ Renal tubular epithelial cells (RTECs) are the core components of the renal tubular system, occupy most of the cell volume, and play an important role in the overall structure and function of the kidney. Studies have shown that RTECs contain a large number of mitochondria, which are easily affected by pathological conditions such as hypoxia and ischemia.^[^ ^6^, ^7^ ^]^ Mitochondrial dysfunction of RTECs prompts their transformation into a secretory phenotype, resulting in the release of pro‐inflammatory mediators (such as pro‐inflammatory cytokines, chemokines, growth factors, peroxides, and C‐reactive proteins), which exacerbate the inflammatory microenvironment and promote the progression of renal fibrosis.^[^ ^8^, ^9^, ^10^ ^]^

Pyruvate carboxylase (PC) is an important catalytic enzyme located within the mitochondria and is widely expressed in kidney, liver, and islet cells.^[^ ^11^, ^12^ ^]^ PC's main function is to catalyze the conversion of pyruvate to oxaloacetic acid, providing an important substrate for gluconeogenesis and the mitochondrial tricarboxylic acid (TCA) cycle and participating in the production of glucose, ATP, and nicotinamide adenine dinucleotide phosphate. Moreover, it can provide intermediates for a variety of biological metabolic pathways, such as fat metabolism, amino acid metabolism, and the urea cycle, playing an important role in the normal life activities of mammals.^[^ ^13^, ^14^, ^15^ ^]^ PC is correlated with mitochondrial damage and the metabolism; however, the relationship between PC and the occurrence and development of kidney fibrosis have not been reported, and its regulatory mechanism remains unclear.

In this study, we found that PC was significantly decreased in RTECs of mouse and human fibrotic kidneys. Conditional knockout of *Pcx* in RTECs exacerbates unilateral ureteral obstruction (UUO)‐induced renal fibrosis. Mechanistically, deficiency of PC reduces sulfide:quinone oxidoreductase (SQOR) stability and accelerates mitochondrial damage, promoting mtDNA leakage and cGAS‐STING signaling activation, which in turn, leads to glycolysis increase and renal fibrosis. Our findings reveal a novel pathogenetic mechanism of renal fibrosis, which has implications for potential targeted therapeutic interventions.

## 2 Results

### 2.1 PC Was Significantly Decreased in Mouse and Human Fibrotic Kidneys

**Figure 1:**
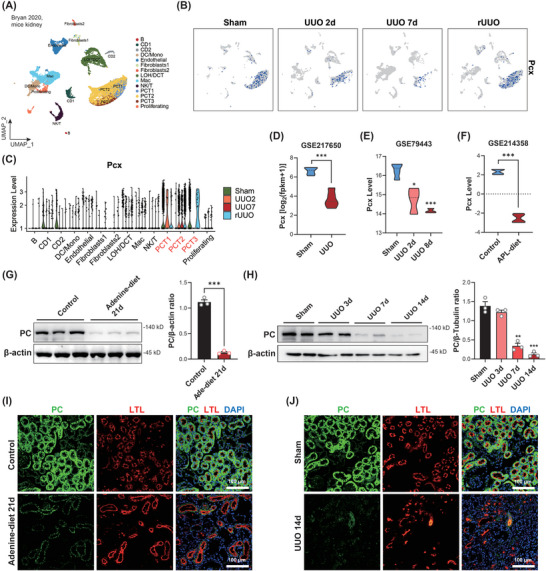
Pyruvate carboxylase (PC) was significantly decreased in various animal renal fibrotic models. A) Composition and distribution of single cells from GSE140023. B) Uniform manifold approximation and projection (UMAP) and C) violin plot of the GSE140023 dataset showing the *Pcx* expression pattern in mouse kidneys that underwent sham or unilateral ureteral obstruction (UUO) operations. The mRNA levels of D,E) *Pcx* in mouse sham or UUO kidneys and F) in allopurinol diet rats and wildtype (WT) controls. G) Western blot analysis and densitometric quantification of PC expression in kidney cortical tissues from control diet mice (*n* = 3) and mice subjected to 21 days of adenine diet (*n* = 3). H) Western blot analysis and densitometric quantification of PC expression in kidney cortical tissues from WT mice (*n* = 3) and UUO mice (*n* = 3). I) Immunofluorescence for PC/LTL in control kidneys and those of mice subjected to 21 days of adenine diet. Scale bar, 100 µm (*n* = 4). J) Immunofluorescence for PC/lotus tetragonolobus lectin (LTL) in sham kidneys and those of mice subjected to 14 days of UUO. Scale bar: 100 µm (*n* = 4). Results are expressed as mean ± SEM. *P < 0.05; **P < 0.01; and ***P < 0.001.

To clarify the expression pattern of PC in kidney fibrosis, we downloaded a single‐cell RNA sequencing dataset (GSE140023) of mouse kidneys, which was classified into four groups: sham surgery (Sham), UUO for 2 days (UUO 2 days), UUO for 7 days, and reversal after 7 days of UUO with the release of ureteral ligation (R‐UUO) (**Figure** **1**A). Our analysis of the PC homologous gene *Pcx* in mice revealed that *Pcx* was highly expressed in the tubular epithelial cells of normal mouse kidneys (Figure S1A,B, Supporting Information). Furthermore, the expression of *Pcx* decreased as the duration of UUO increased but rebounded after the reversal of ureteral obstruction (Figure 1B,C; Figure S1B, Supporting Information). Transcriptomic data of mouse or rat's kidneys (GSE217650, GSE79443, and GSE214358) showed that the expression level of *Pcx* was significantly downregulated in renal fibrosis models induced by UUO or an allopurinol diet compared to normal controls (Figure 1D–F).

**Figure 2:**
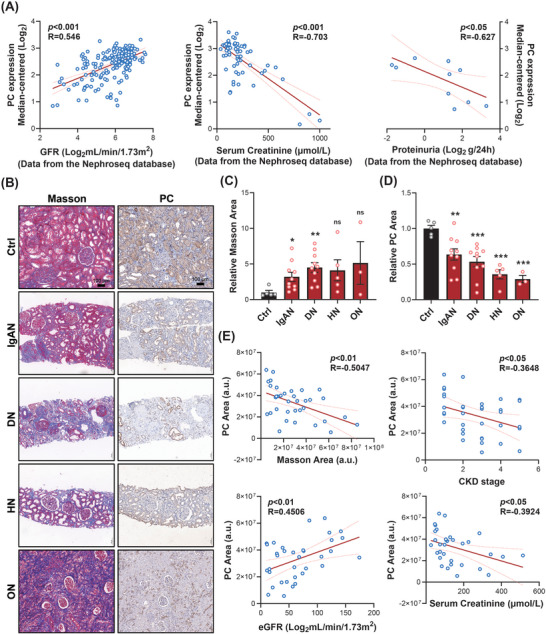
Pyruvate carboxylase (PC) expression correlated with renal function and fibrosis in patients with chronic kidney disease (CKD). A) Correlation analysis of PC and glomerular filtration rate (GFR), serum creatinine, and proteinuria with the data of patients with CKD from the Nephroseq database. B) Representative images for Masson's trichrome and immunohistochemical staining of PC expression in renal biopsy specimens from patients with IgA nephropathy (IgAN, *n* = 10), diabetic nephropathy (DN, *n* = 10), hypertensive nephropathy (HN, *n* = 5), and obstructive nephropathy (ON, *n* = 3). Statistical graphs for C) Masson's trichrome and D) immunohistochemical staining. E) Correlation analysis of PC and Masson staining area, CKD stage, estimated GFR, and serum creatinine from subjects with IgAN, DN, HN, and ON. Scale bar: 100 µm. Results are expressed as mean ± SEM. *P < 0.05; **P < 0.01; and ***P < 0.001.

Subsequently, we subjected C57 mice to adenine diet‐induced renal fibrosis for 21 days and observed a significant reduction in PC expression (Figure 1G). Samples were collected on days 3, 7, and 14 to establish the UUO renal fibrosis model. Western blot analysis revealed that PC protein expression in the mouse kidneys progressively decreased as UUO progressed (Figure 1H). Immunofluorescence staining of the kidneys demonstrated that PC was highly expressed in tubular epithelial cells (marked with the anti‐fluorescein‐labeled lotus tetragonolobus lectin [LTL] antibody) in normal mice and was significantly downregulated in the UUO or adenine diet‐induced model (Figure 1I,J; Figure S1C,D, Supporting Information). Moreover, we detected PC expression in patients with CKD. Data from the Nephroseq database showed that renal tissue PC expression is positively correlated with glomerular filtration rate (GFR) (mL min^−1^/1.73 m^2^) (*N* = 186, *R* = 0.546, and *p* < 0.001), negatively correlated with serum creatinine levels (*N* = 62, *R* = −0.703, and *p* < 0.001), and negatively correlated with 24‐h urinary protein levels (*N* = 11, *R* = −0.627, and *p* < 0.05) in patients with CKD (**Figure** **2**A). Renal biopsy samples from patients with CKD (TableS1, Supporting Information) revealed that PC expression in renal tubules was downregulated in patients with multiple renal tubulointerstitial fibrosis diseases, including IgA nephropathy (IgAN), diabetic nephropathy (DN), hypertensive neuropathy (HN), and obstructive nephropathy (ON) (Figure 2B–D). Notably, the level of PC in the renal tubules was negatively correlated with Masson staining (*R* = −0.505 and *p* < 0.01), negatively correlated with CKD staging (*R* = −0.365 and *p* < 0.05), positively correlated with patients’ eGFR (*R* = 0.451 and *p* < 0.01), and negatively correlated with patients' serum creatinine levels (*R* = −0.392 and *p* < 0.05) (Figure 2E). These results indicate that PC may play an important role in kidney fibrosis.

### 2.2 *Pcx* Deficiency Induces Spontaneous Kidney Fibrosis

**Figure 3:**
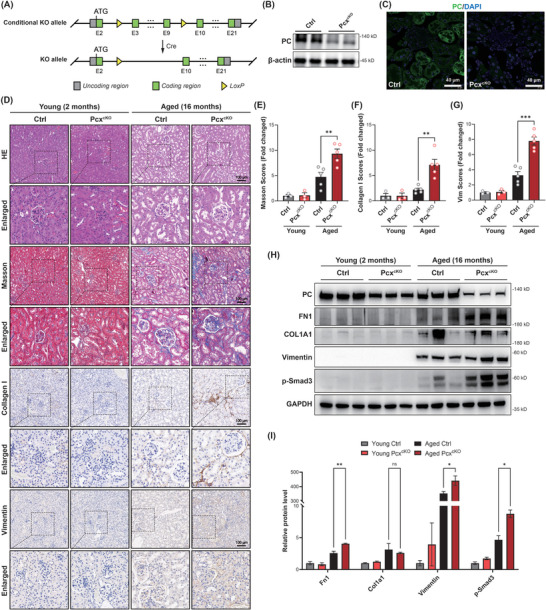
*Pcx* deficiency induces spontaneous kidney fibrosis. A) Schematic representation illustrating the genetic approach used to generate *Pcx* conditional knockout mice (*Pcx* ^cKO^). B) Western blot analysis and C) immunofluorescence detected the pyruvate carboxylase (PC) protein levels in control mouse (*Pcx* ^flox/flox^) and 2‐month‐old *Pcx* ^cKO^ mouse kidney tissues. D) Representative images and statistical graphs for hematoxylin and eosin (HE), E) Masson's trichrome, and immunohistochemical staining of F) collagen I and G) vimentin expression in renal tissues from control and *Pcx* ^cKO^ mice aged 2‐ or 16‐months‐old (*n* = 5/ea.). Dashed squares indicate the enlarged regions. H,I) Western blot analysis and densitometric quantification of PC, FN1, COL1A1, vimentin, and p‐Smad3 in kidney tissues from control and *Pcx* ^cKO^ mice at 2 or 16 months (*n* = 3/ea.). Scale bar, white, 40 µm; black, 100 µm. Results are expressed as mean ± SEM. *P < 0.05; **P < 0.01; and ***P < 0.001.

To further explore the relevance and mechanism of PC in renal fibrosis, we used CRISPR‐Cas9 technology to create a model of conditionally knocked‐out PC in tubular epithelial cells of mice (*Pcx* ^flox/flox^ *;Cdh16‐Cre*, *Pcx* ^cKO^) (**Figure** **3**A; Figure S2A, Supporting Information). Western blot analysis and immunofluorescence staining revealed the absence of PC expression in the tubules of *Pcx* ^cKO^ mice, indicating successful model construction (Figure 3B,C). We compared the appearance, body weight, and kidney histology of 2‐month‐old *Pcx* ^cKO^ mice with those of control mice (*Pcx* ^flox/flox^) and found that 2‐month‐old cKO mice did not exhibit obvious defects or renal dysfunction (Figure S2B–D, Supporting Information). Subsequently, we collected renal tissues from 2‐month‐old and 16‐month‐old *Pcx* ^cKO^ and control mice. HE, Masson's trichrome, collagen I, and vimentin immunohistochemical staining showed that *Pcx* ^cKO^ mice exhibited significant interstitial fibrosis at 16 months of age compared to control mice (Figure 3D–G). RT‐PCR and western blot analyses showed that the mRNA and protein expression levels of markers such as FN1, collagen I, and vimentin were significantly elevated in the kidneys of 16‐month‐old *Pcx* ^cKO^ mice compared to those in control mice (Figure 3H,I; Figure S2E, Supporting Information). This suggests that *Pcx* ^cKO^ mice exhibited spontaneous renal fibrosis.

### 2.3 Conditional Knockout of PC in Tubular Epithelial Cells Exacerbates UUO‐Induced Renal Fibrosis

**Figure 4:**
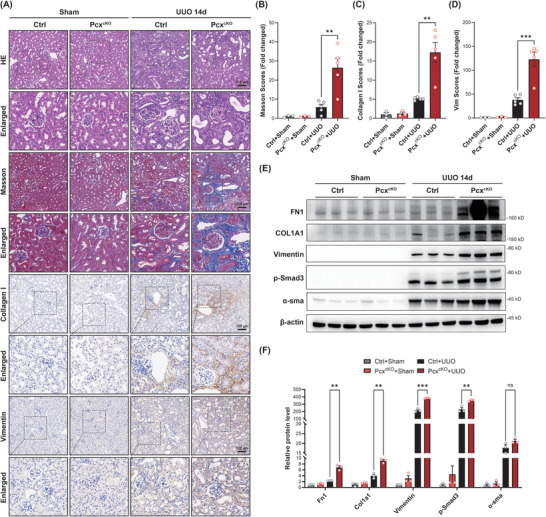
Conditional knockout of pyruvate carboxylase (PC) in tubular epithelial cells exacerbates unilateral ureteral obstruction (UUO)‐induced renal fibrosis. A) Representative images and statistical graphs for hematoxylin and eosin (HE), B) Masson's trichrome, and immunohistochemical staining of C) collagen I and D) vimentin expression in renal tissues from control and *Pcx* ^cKO^ mice subjected to sham operation or 14 days after the UUO operation (*n* = 5/ea.). Dashed squares indicate the enlarged regions. E,F) Western blot analysis and densitometric quantification of FN1, COL1A1, vimentin, p‐Smad3, and α‐SMA in kidney tissues from control and *Pcx* ^cKO^ mice subjected to sham operation or 14 days after the UUO operation (*n* = 3/ea.). Scale bar: 100 µm. Results are expressed as mean ± SEM. **P < 0.01 and ***P < 0.001.

Next, we used 2‐month‐old *Pcx* ^cKO^ mice to study the effect of PC deficiency on the progression of renal fibrosis following UUO. Serum renal function tests showed that the levels of serum creatinine, uric acid, and urea nitrogen were significantly increased in UUO‐treated mice, indicating successful UUO modeling (Figure S3A–C, Supporting Information). *Pcx* ^cKO^ mice exhibited significantly higher levels of serum creatinine and uric acid compared with flox control mice (Figure S3A–C, Supporting Information). Histological analysis revealed that both *Pcx* ^cKO^ and flox control mice showed significant tubular dilation, atrophy, and interstitial inflammation in the obstructed kidneys compared to the contralateral kidney in sham surgery. However, the degree of fibrosis was much more severe in *Pcx* ^cKO^ mice than in the flox controls (**Figure** **4**A). Masson's trichrome, collagen I, and vimentin immunohistochemical staining indicated a significant increase in the accumulation of extracellular matrix (ECM) in the obstructed kidneys of *Pcx* ^cKO^ mice (Figure 4A–D). Consistent with the pathological and serological findings, we observed a significant increase in the protein expression levels of markers such as FN1, collagen I, and vimentin in the kidneys of *Pcx* ^cKO^ mice (Figure 4E,F; Figure S3D, Supporting Information).

### 2.4 PC Deficiency Can Exacerbate the Fibrosis of HK‐2 Cells Induced by TGF‐β1

**Figure 5:**
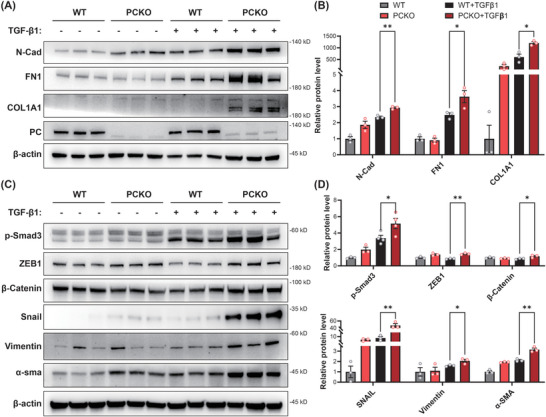
Pyruvate carboxylase (PC) deficiency can exacerbate the fibrosis of HK‐2 cells induced by TGF‐β1. HK‐2 cells underwent stable knock‐out of control or PC (WT or PCKO) genes and were stimulated with TGF‐β1 for 24 h (15 ng mL^−1^). A,B) Western blot analysis and densitometric quantification of PC and extracellular matrix components (N‐CAD, FN1, and COL1A1) expression (*n* = 3/ea.). C,D) Western blot analysis and densitometric quantification of Wnt/β‐Catenin/Snail and partial epithelial–mesenchymal transition (EMT) pathways (p‐SMAD3, ZEB‐1, β‐Catenin, SNAIL, vimentin, and α‐SMA) expression (*n* = 3/ea.). Results are expressed as mean ± SEM. *P < 0.05 and **P < 0.01. WT, wildtype.

Subsequently, we utilized CRISPR‐Cas9 technology to create a stable knockout of PC in human tubular epithelial cells HK‐2. The cells were stimulated with TGF‐β1 for 24 h and subjected to western blot analysis. The results showed that the expression of ECM components such as N‐CAD, FN1, and COL1A1 was significantly increased in the PC knockout (PCKO) group under TGF‐β1 stimulation compared to the control group. This suggests that the knock‐out of PC may exacerbate ECM production and deposition induced by TGF‐β1 in HK‐2 cells (**Figure** **5**A,B; Figure S4, Supporting Information). In addition, the examination of molecules related to the Wnt/β‐Catenin/Snail and partial epithelial–mesenchymal transition pathways, including p‐SMAD3, ZEB‐1, β‐Catenin, SNAIL, vimentin, and α‐SMA, revealed a significant increase in the expression of fibrosis pathway molecules in PCKO cells compared to those in the control (Figure 5C,D). These results suggest that the lack of PC may exacerbate TGF‐β1‐induced ECM deposition and fibrosis in HK‐2 cells.

### 2.5 PC Knockout Enhanced Glycolysis and Fibrotic Changes

**Figure 6:**
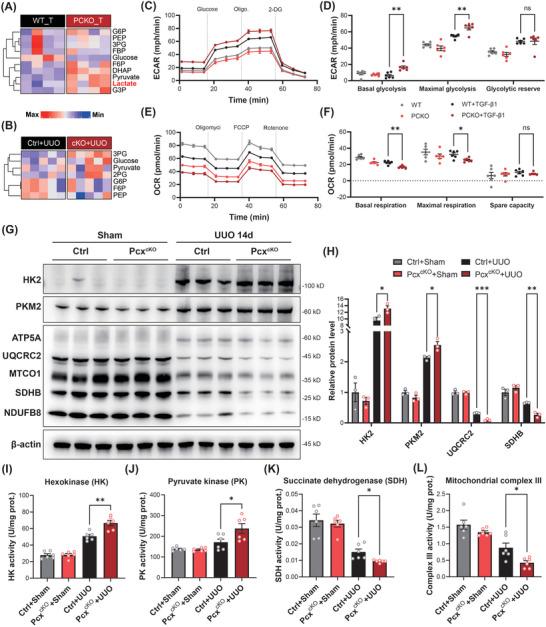
Pyruvate carboxylase (PC) knockout increased glycolysis levels. A,B) Targeted metabolomics showed the metabolites of glycolysis in HK‐2 cells of the WT+TGF‐β1 and PCKO+TGF‐β1 groups and mouse kidney of *Pcx* ^flox/flox^+UUO and *Pcx* ^cKO^+UUO groups. The color in the heatmaps from blue to red shows the progression from low expression to high expression, respectively. C) Mitochondrial extracellular acidification rate (ECAR) in HK‐2 cells of different groups (*n* = 6/ea.). D) Basal glycolysis, maximal glycolysis, and glycolytic reserve in HK‐2 cells of different groups (*n* = 6/ea.). E) Mitochondrial oxygen consumption rate (OCR) in HK‐2 cells of different groups (*n* = 6/ea.). F) Basal respiration, maximal respiration, and spare respiratory capacity in HK‐2 cells of different groups (*n* = 6/ea.). G,H) Western blot analysis and densitometric quantification of HK2, PKM2, ATP5A, UQCRC2, MTCO1, SDHB, and NUDFB8 kidney tissues from control and *Pcx* ^cKO^ mice subjected to sham operation or 14 days after the UUO operation (*n* = 3/ea.). I–L) Enzymatic activity assay results of hexokinase (HK), pyruvate kinase (PK), succinate dehydrogenase (SDH), and mitochondrial complex III in kidney tissues from control and *Pcx* ^cKO^ mice subjected to sham operation or 14 days after the UUO operation (*n* = 6/ea.). Results are expressed as mean ± SEM. *P < 0.05; **P < 0.01; and ***P < 0.001; ns, non‐significant. WT, wildtype.

To further explore the mechanism of PC deficiency‐induced fibrosis, we performed metabolomics detection of PCKO and control both in vivo and in vitro. The metabolomic results showed that glycolytic metabolites (such as glucose‐6‐phosphate, fructose‐6‐phosphate, dihydroxyacetone phosphate, and glyceraldehyde 3‐phosphate) were significantly upregulated in the PCKO+TGF‐β1 group or *Pcx* ^cKO^+UUO mice, indicating that the absence of PC disrupted mitochondrial metabolic balance and enhanced glycolysis levels (**Figure** **6**A,B; Figure S5A,B, Supporting Information). However, the level of gluconeogenesis did not show significant changes after PC knockout (Figure S5C, Supporting Information). Seahorse analysis confirmed that the extracellular acidification rate (ECAR) was much higher in the PCKO+TGF‐β1 group than in controls; while, the oxygen consumption rate (OCR) was significantly decreased in PCKO cells under TGF‐β1 treatment (Figure 6C–F).

Subsequently, we validated these findings at the overall level using western blot analysis and enzymatic assays. The results showed that key glycolytic enzymes such as hexokinase (e.g., HK2) and pyruvate kinase (e.g., PKM2) were significantly upregulated after UUO, whereas key oxidative phosphorylation enzymes such as mitochondrial complex III (e.g., UQCRC2) and succinate dehydrogenase (e.g., SDHB) were significantly downregulated in UUO‐induced *Pcx* ^cKO^ mice compared to control mice, both in expression levels (Figure 6G,H) and enzymatic activities (Figure 6I–L). Besides, considering that oxaloacetate (OAA) is the primary product of PC, we also assessed the levels of OAA in the UUO animal model. Our results showed that the OAA levels were significantly increased in the kidneys of UUO mice, but no differences were detected between the control and *Pcx* ^cKO^ groups (Figure S5D, Supporting Information). This suggests that PC may influence renal fibrosis through mechanisms other than its downstream product, OAA.

These results collectively demonstrated that PC knockout can enhance glycolysis induced by TGF‐β1 or UUO, leading to fibrotic changes in RTECs.

### 2.6 PC Knockout Induced Mitochondrial Damage and Activated the cGAS‐STING Pathway

Furthermore, we analyzed RNA transcriptome sequencing of PCKO and control HK‐2 cells before and after TGF‐β1 stimulation. Differential genes were selected based on the criteria of Log_2_|Fold‐Changed (FC)|>1.0 and adjusted *p* < 0.05, and their expression across the four groups is displayed in a heatmap (Figure S6A and Table S3, Supporting Information). Gene Ontology (GO) biological process enrichment analysis showed a significant increase in the expression of ECM‐encoding genes after TGF‐β1 stimulation (Figure S6B, Supporting Information). In addition, PCKO cells exhibited a significantly higher degree of ECM gene expression post‐TGF‐β1 stimulation compared to the wild‐type (WT) TGF‐β1 stimulated group (Figure S6C, Supporting Information). Gene set enrichment analysis (GSEA) indicated significant enrichment of pro‐inflammatory and pro‐fibrotic pathways in the PCKO+TGF‐β1 stimulated group, as demonstrated in the heatmap, with marked elevation in several fibrosis‐related genes (Figure S6D, Supporting Information).

**Figure 7:**
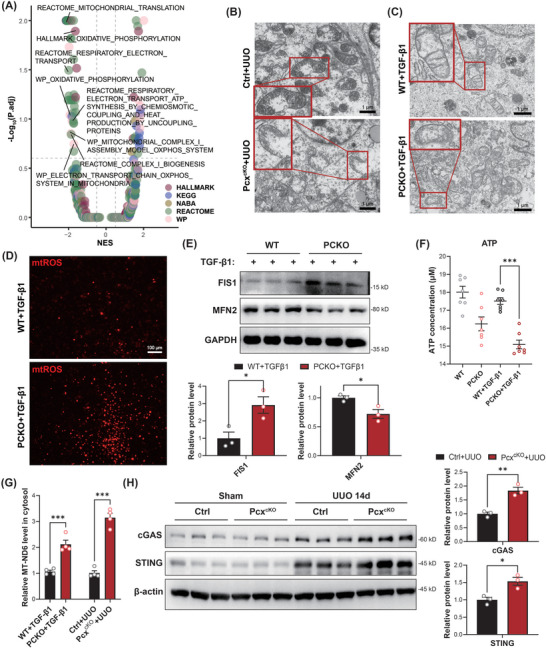
Pyruvate carboxylase (PC) knockout‐induced mitochondrial damage and activated cGAS‐STING pathway. A) Volcano plot of the profile of the different gene sets (color coded) after performing GSEA on the RNA‐Seq landscape of the PCKO+TGF‐β1 cells (*n* = 3) compared to WT+TGF‐β1 cells (*n* = 3). Adjusted *p*‐value (p.adj) and normalized enrichment score (NES) result from the GSEA. B) Representative electron micrograph of mitochondria in kidney tissues from control and *Pcx* ^cKO^ mice subjected to sham operation or 14 days after the UUO operation. C) Representative electron micrograph of mitochondria in HK‐2 cells of the WT+TGF‐β1 and PCKO+TGF‐β1 groups. D) Representative images of mitochondrial ROS under fluorescence microscope of HK‐2 cell of WT+TGF‐β1 and PCKO+TGF‐β1 groups. E) Western blot analysis and densitometric quantification of FIS1 and MFN2 in HK‐2 cells of the WT+TGF‐β1 and PCKO+TGF‐β1 groups (*n* = 3/ea.). F) ATP assay of HK‐2 cells of different groups (*n* = 7/ea.). G) Real‐time polymerase chain reaction (RT‐PCR) revealed the mRNA levels of mtDNA (MT‐ND6) in the WT+TGF‐β1 and PCKO+TGF‐β1 groups, as well as in the *Pcx* ^flox/flox^+UUO and *Pcx* ^cKO^+UUO groups (*n* = 4/ea.). H) Western blot analysis and densitometric quantification of cGAS and STING kidney tissues from control and *Pcx* ^cKO^ mice subjected to sham operation or 14 days after the UUO operation (*n* = 3/ea.). Scale bar, 1 µm. Results are expressed as mean ± SEM. *P < 0.05; **P < 0.01; and ***P < 0.001. GSEA, Gene set enrichment analysis; WT, wildtype.

Beyond fibrosis‐related pathways, GSEA enrichment analysis also indicated weakening of the respiratory chain pathway in PCKO+TGF‐β1 stimulated cells, suggesting exacerbated mitochondrial damage (**Figure** **7**A). To verify this, we examined UUO mouse kidneys and WT and PCKO HK‐2 cells under TGF‐β1 stimulation using electron microscopy. As shown in Figure 7B, the mitochondrial membranes were much ruptured, with mitochondria atrophied and mitochondrial ridges decreased or even having disappeared in *Pcx* ^cKO^ mouse kidneys under UUO. Similarly, cells in the PCKO+TGF‐β1 group showed statistically significant mitochondrial shrinkage and reduced mitochondrial cristae compared to those in the WT+TGF‐β1 stimulated group (Figure 7C; Figure S7A, Supporting Information). ROS fluorescence staining and JC‐1 mitochondrial membrane potential tests also showed that the mitochondrial ROS levels were significantly elevated and mitochondrial membrane potentials decreased in the PCKO+TGF‐β1 group compared to the WT+TGF‐β1 group (Figure 7D; Figure S7B,C, Supporting Information). At the molecular level, we detected the expression of the mitochondrial fission protein fission 1 (FIS1) and mitochondrial fusion protein mitofusin 2 (MFN2) using western blot analysis. The results showed that MFN2 expression was significantly downregulated and FIS1 expression was significantly elevated in the PCKO+TGF‐β1 stimulated cells compared to the WT+TGF‐β1 group (Figure 7E). ATP content measurement revealed that ATP levels were significantly lower in PCKO+TGF‐β1 cells compared to WT+TGF‐β1 cells, suggesting a reduction in energy production (Figure 7F).

Previous studies have shown that mitochondrial damage leads to mtDNA leakage into the cytoplasm, which may activate the DNA sensor cGAS‐STING and its downstream signaling pathways. Hence, we detected the levels of mtDNAs (including *ND6*, *CytB*, *RNR2*, and *CO1*) in the cytosol and found that all of these mtDNAs were significant increased in the cytosol of *Pcx* ^cKO^+UUO mice/PCKO+TGF‐β1 cells compared with the control group (Figure 7G; Figure S7D, Supporting Information), indicating leakage of mtDNA into the cytosol. Besides, the western blot analysis results indicated that, compared to the control UUO mice, *Pcx* ^cKO^+UUO mice exhibited significantly increased levels of cGAS and STING proteins in the kidneys. These findings suggest that PC knockout induces mitochondrial damage and mtDNA leakage into the cytoplasm, triggering the activation of the cGAS‐STING signaling pathway.

### 2.7 PC Deficiency Reduces SQOR Stability and Accelerates Mitochondrial Damage

**Figure 8:**
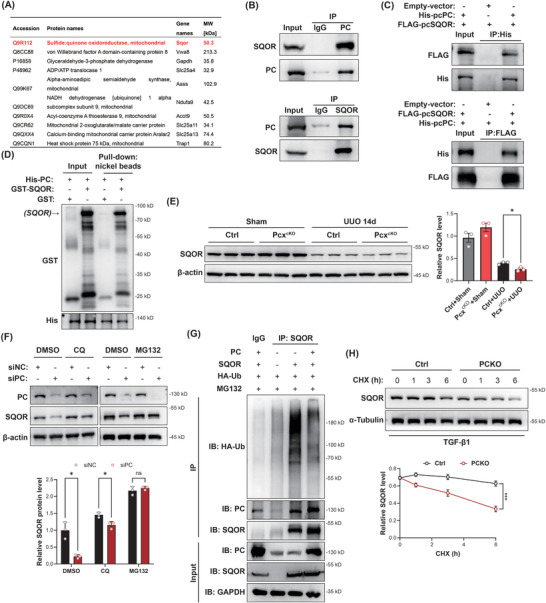
Pyruvate carboxylase (PC) deficiency reduces sulfide:quinone oxidoreductase (SQOR) stability. A) Top ten candidate interacting proteins. B) Co‐immunoprecipitation analysis investigating the interaction of endogenous PC with SQOR in HK‐2 cells. C) Co‐immunoprecipitation analysis investigating the interaction of exogenous His‐PC with FLAG‐SQOR in 293T cells. D) Glutathione *S*‐transferase (GST)‐tagged SQOR and His‐tagged PC were purified from *E. coli*. The beads binding the target proteins were incubated with GST or GST‐SQOR fusion proteins, and the pull‐down proteins were detected with anti‐GST antibody with immunoblotting. E) Western blot analysis and densitometric quantification of SQOR kidney tissues from control and *Pcx* ^cKO^ mice subjected to sham operation or 14 days after the UUO operation (*n* = 3/ea.). F) Western blot analysis and densitometric quantification of PC and SQOR in siNC or siPC infected HK‐2 cells treated with 10 µm CQ or 10 µm MG132 for 6 h (*n* = 3/ea.). G) Co‐immunoprecipitation assay for the ubiquitination of SQOR in HEK293 cells transfected with pcPC and ubiquitin‐expressing plasmids and treated with MG132. H) Cycloheximide (CHX) chase assay for SQOR in WT and PCKO HK‐2 cells treated with CHX (500 µg mL^−1^) for the indicated time points (*n* = 3/ea.). Results are expressed as mean ± SEM. *P < 0.05 and ***P < 0.001.

Subsequently, we investigated the mechanism through which PC regulates mitochondrial damage‐induced mtDNA release. To elucidate the molecular mechanisms involved, we performed LC‐MS/MS proteomic analysis on PC immunoprecipitates from mouse kidney tissues, identifying 22 potential interacting proteins with a score of 100 or higher. Among these, SQOR ranked first (**Figure** **8**A). SQOR is a key enzyme involved in sulfide metabolism and plays a crucial role in regulating mitochondrial structure and function.^[^ ^16^ ^]^ It has been reported that SQOR deficiency can induce mitochondrial membrane lipid peroxidation, leading to mitochondrial damage and ferroptosis. Our experiments further corroborate this as silencing SQOR in HK‐2 cells followed by treatment with the ferroptosis inducer erastin resulted in western blot analysis, showing a significant downregulation of the ferroptosis marker GPX4 (Figure S8A, Supporting Information). In addition, silencing SQOR exacerbated the increase in FN1 expression induced by TGF‐β1 stimulation (Figure S8B, Supporting Information). These findings suggest that downregulation of SQOR expression in RTECs may lead to mitochondrial membrane lipid peroxidation, mitochondrial rupture, and subsequent mitochondrial damage.

CO‐IP and pull‐down assays both demonstrated that PC protein could interact with SQOR (Figure 8B–D; Figure S8C, Supporting Information). Immunofluorescence further demonstrated the colocalization of PC and SQOR in cells (Figure S8D, Supporting Information). Western blot results showed that SQOR expression was significantly downregulated in the kidneys of *Pcx* ^cKO^+UUO mice compared to control UUO mice; while, the real‐time PCR result showed no significant change (Figure 8E; Figure S8E, Supporting Information). Moreover, treatment with the proteasome inhibitor MG132, but not the autophagy inhibitor CQ, abrogated the downregulation of SQOR induced by PC silencing in HK‐2 cells (Figure 8F), indicating that PC inhibits proteasomal degradation of SQOR. Under stimulation with the MG132, co‐transfection of SQOR and PC significantly reduced their binding to ubiquitin (Figure 8G). In addition, CHX chase experiments further revealed that PC knockout decreased the half‐life of SQOR protein in HK‐2 cells (Figure 8H). These results indicate that PC deficiency reduces its interaction with SQOR, thereby promoting the degradation of SQOR via the ubiquitin–proteasome pathway under pathological conditions of renal fibrosis. This, in turn, exacerbates mitochondrial damage and mtDNA release.

### 2.8 Increased PC Alleviates Metabolic Reprogramming and Renal Fibrosis After UUO

**Figure 9:**
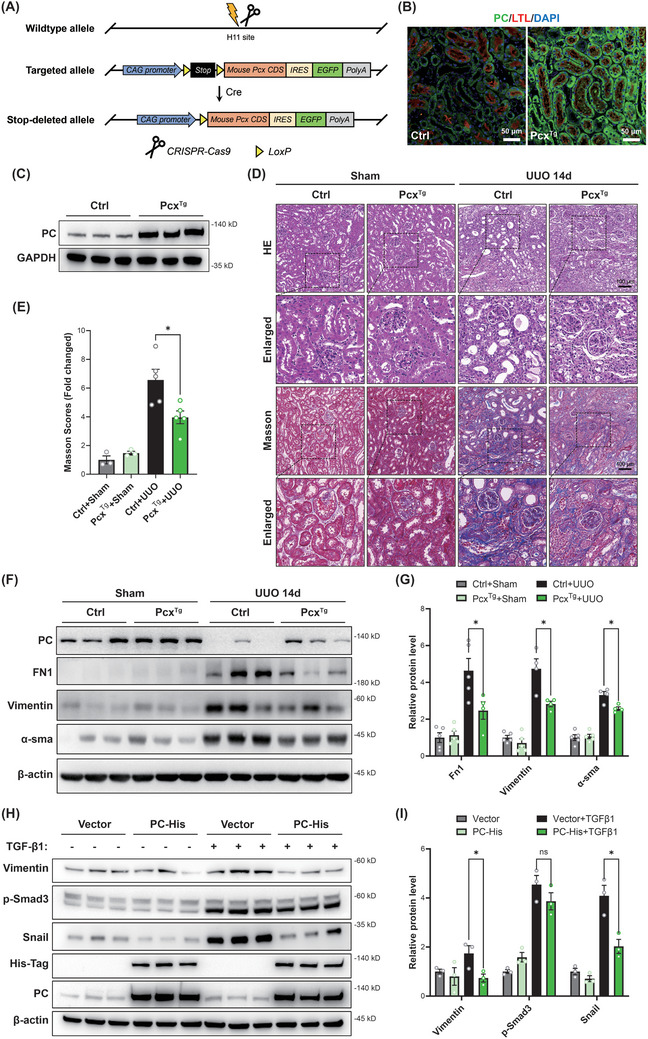
Pyruvate carboxylase (PC) alleviates metabolic reprogramming and renal fibrosis after unilateral ureteral obstruction (UUO). A) Schematic representation illustrating the genetic approach used to generate *Pcx* conditional transgenic mice (*Pcx* ^Tg^). B) Immunofluorescence and C) western blot analysis detected the PC protein levels in control mouse (*Pcx* ^flox/flox^) and 2‐month‐old *Pcx* ^Tg^ mouse kidney tissues. D) Representative images and statistical graphs for HE and E) Masson's trichrome in renal tissues from control and *Pcx* ^Tg^ mice subject to sham operation or 14 days after UUO operation (*n* = 5/ea.). F,G) Western blot analysis and densitometric quantification of PC, FN1, vimentin, and α‐SMA in kidney tissues from control and *Pcx* ^Tg^ mice subjected to sham operation or 14 days after the UUO operation (*n* = 5/ea.). H,I) Western blot analysis and densitometric quantification of vimentin, p‐Smad3, Snail, His‐tag, and PC in HK‐2 cells transfected with vector plasmids or PC‐His expression plasmids with or without TGF‐β1 stimulation (*n* = 3/ea.). Scale bar, white, 50 µm; black, 100 µm. Results are expressed as mean ± SEM. *P < 0.05.

Next, we evaluated the potential of PC to ameliorate renal fibrosis after UUO. CRISPR‐Cas9 technology was used to create tubular epithelial cell‐specific *Pcx* overexpressing (*Pcx* ^Tg^) mice (**Figure** **9**A **;** Figure S9A,B, Supporting Information). Immunofluorescence staining and western blot analysis showed PC overexpression in the tubules of *Pcx* ^Tg^ mice, indicating successful model construction (Figure 9B,C). We compared the kidney phenotypes of four experimental groups: WT littermates with sham operation (Ctrl+Sham), *Pcx* ^Tg^ mice with sham operation (*Pcx* ^Tg^+Sham), WT littermates with 14 days of UUO operation (Ctrl+UUO), and *Pcx* ^Tg^ mice with 14 days of UUO operation (*Pcx* ^Tg^+UUO). *Pcx* ^Tg^ mice exhibited significantly lower levels of serum creatinine and uric acid compared with flox control mice (Figure S9C–E, Supporting Information). HE and Masson's trichrome staining indicated lower accumulation of ECM in the obstructed kidneys of *Pcx* ^Tg^ mice (Figure 9D,E). Western blot and RT‐PCR analyses revealed that the expression levels of fibrotic markers such as FN1, vimentin, and α‐SMA were significantly down‐regulated in the *Pcx* ^Tg^+UUO group (Figure 9F,G). Similarly, transfection of PC‐expression plasmids in HK‐2 cells rescued the high expression of vimentin and Snail caused by TGF‐β1 stimulation (Figure 9H,I).

Taken together, these findings indicate that PC alleviates renal fibrosis after UUO, ultimately recovering impaired kidney function.

## 3 Discussion

**Figure 10:**
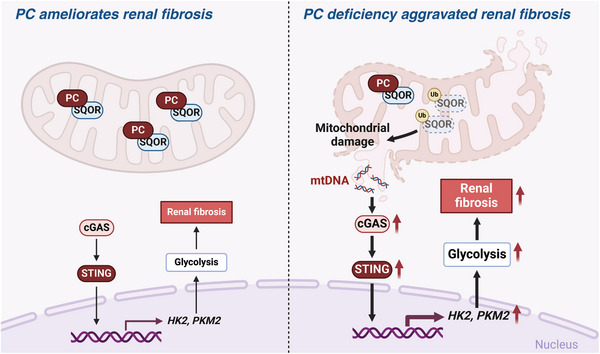
Scheme depicting the mechanism of how pyruvate carboxylase (PC)‐deficiency exacerbates renal fibrosis. Under fibrotic conditions, the downregulation of PC in renal tubular epithelial cells (RTECs) reduces its interaction with sulfide:quinone oxidoreductase (SQOR), increasing the ubiquitination and degradation of SQOR. This leads to mitochondrial morphological and functional disruption, increased mtDNA release, activation of the cGAS‐STING pathway, elevated glycolysis levels, and ultimately, promotion of renal fibrosis. Consequently, depleted PC levels significantly increase the deposition of collagen and fibronectin, leading to aggravated kidney fibrosis. Figure created in BioRender. Huang, H. (2025) https://BioRender.com/q76×687.

PC catalyzes the carboxylation of pyruvate to OAA, serving as a critical component in gluconeogenesis and an intermediate in the TCA cycle.^[^ ^13^, ^17^ ^]^ Studies have reported that PC plays a vital role in processes such as insulin secretion, fat synthesis, and neurotransmitter biosynthesis, allowing it to be a potential target for diseases such as obesity, diabetes, viral infections, and cancer.^[^ ^18^, ^19^, ^20^ ^]^ However, despite it being crucial for energy metabolism, PC's function in renal diseases remains unknown. In this study, we provided clinical and molecular evidence suggesting that PC deficiency in RTECs may contribute to CKD and renal fibrosis (**Figure** **10**). Our findings suggest a possible causal relationship between downregulated PC expression and renal fibrosis.

In Cappel et al.’s study, liver‐specific conditional PC knockout mice fed a high‐fat diet (HFD) had a threefold increase in the aspartate transaminase/alanine transaminase ratio compared with HFD control mice, indicating exacerbated liver cell damage. In addition, after 12 weeks of HFD, liver‐specific PC knockout mice exhibited significantly increased expression of inflammation‐and cell death‐related genes compared to HFD controls,^[^ ^21^ ^]^ suggesting that PC knockout in the liver can induce oxidative stress and inflammatory responses, exacerbating liver damage caused by HFD. Moreover, PC is crucial for brain metabolism. Several studies have shown that neurotransmitters released by brain neurons, such as glutamate, can be replenished through the glutamate–glutamine neurotransmitter cycle and de novo synthesis of glutamate, mediated by PC.^[^ ^22^, ^23^, ^24^ ^]^ Imbalance in PC in the brain is linked to various psychiatric diseases, including bipolar disorder and epilepsy.^[^ ^25^, ^26^ ^]^

In this study, we knocked out PC in RTECs and explored its role in mitochondrial damage and metabolism and its relationship with the development and progression of renal fibrosis. Our in vitro and in vivo experiments revealed that PC knockout significantly increased the FIS1/MFN2 ratio and mitochondrial ROS levels in RTECs under TGF‐β1 stimulation, significantly downregulating the mitochondrial membrane potential and affecting morphology (Figure 7). Notably, through CO‐IP experiments, we further confirmed that PC can interact with SQOR, stabilizing SQOR expression levels. SQOR catalyzes the conversion of sulfide to persulfide in the mitochondria, thereby reducing toxic sulfide concentrations, preventing mitochondrial membrane peroxidation, maintaining mitochondrial integrity, and mitigating ferroptosis.^[^ ^16^, ^27^, ^28^, ^29^, ^30^ ^]^ In our study, we found that PC deficiency mediated the ubiquitination and degradation of SQOR, which disrupted mitochondrial morphology and function. This mitochondrial damage led to mtDNA leakage and activation of the cGAS‐STING pathway. In recent years, the cGAS‐STING signaling pathway has been recognized as a crucial cytoplasmic DNA sensing pathway with significant roles in cancer, infections, autoimmune diseases, and inflammation.^[^ ^31^, ^32^, ^33^ ^]^ Previous studies have shown that dysregulation of the cGAS‐STING pathway is closely associated with renal fibrosis.^[^ ^33^, ^34^ ^]^ In mouse models, knocking out STING or using STING inhibitors significantly alleviated folic acid‐induced renal tubular injury, inflammation, and renal fibrosis.^[^ ^33^ ^]^ In addition, in patients with CKD, the expression levels of cGAS and STING in the kidneys strongly correlated with renal function and the degree of renal fibrosis,^[^ ^6^ ^]^ suggesting that the activation of the cGAS‐STING pathway has broad significance in the development of renal fibrosis.

On the other hand, the activation of the cGAS‐STING pathway is closely related to cellular glycolysis and metabolic reprogramming. Jiang et al. demonstrated that using STING inhibitors or knocking out STING significantly reversed the glycolysis levels and fibrotic changes in RTECs.^[^ ^35^ ^]^ Mechanistically, STING can increase glycolysis by regulating the hypoxia‐inducible factor HIF‐1α.^[^ ^36^ ^]^ A well‐ordered energy metabolism system is crucial for maintaining the unique structure and physiological functions of the kidney.^[^ ^37^ ^]^ Metabolic reprogramming, particularly the shift from oxidative phosphorylation to glycolysis, has emerged as a key contributor to the pathogenesis of organ fibrosis, including renal fibrosis.^[^ ^38^, ^39^, ^40^ ^]^ In UUO mice, renal glycolysis levels were significantly elevated, and inhibition of RTEC glycolysis using mesenchymal stem cell‐derived exosomes significantly alleviated renal fibrosis.^[^ ^41^ ^]^ In our study, increased expression of glycolytic enzymes, including HK2 and PKM2, was observed in fibrotic kidneys, correlating with interstitial fibrosis and impaired renal function. When we stimulated HK‐2 cells with the glycolytic activator TEPP‐46, the expression of fibrosis markers was significantly increased (Figure S5E, Supporting Information). This provides further evidence that enhanced glycolysis contributes to the progression of renal fibrosis. Wang et al.’s research suggested that glycolytic reprogramming contributes to renal fibrosis through mechanisms beyond energy metabolism. Glycolysis byproducts, such as lactate, may influence gene expression via epigenetic modifications, including histone lactylation, which facilitates inflammation and fibrogenesis in tubular epithelial cells.^[^ ^42^ ^]^

Previous studies have demonstrated that pharmacological inhibition of glycolysis using compounds such as dichloroacetate or shikonin significantly reduced renal fibrosis in UUO mice.^[^ ^43^ ^]^ However, the clinical application of these inhibitors has been limited due to their significant cellular toxicity. Our emerging evidence underscores the central role of PC in glycolytic reprogramming and its involvement in renal fibrosis. In our study, we found that PC knockout in mice increased mtDNA leakage, significantly activated the cGAS‐STING pathway, and subsequently upregulated the expression of glycolytic enzymes such as HK2 and PKM2, promoting renal fibrosis. Conversely, genetically overexpressing PC in mice reversed these effects and ameliorated renal fibrosis. Thus, we speculate that the PC pathway may regulate the activation of the cGAS‐STING signaling pathway, and the upregulation of glycolysis may be the downstream mechanism of PC/SQOR/STING‐mediated renal fibrosis. Based on these findings, the development of small‐molecule compounds targeting PC could minimize potential side effects and offer a promising therapeutic strategy for the treatment of renal fibrosis.

Currently, information about the specific mechanisms underlying metabolic reprogramming from oxidative phosphorylation to glycolysis in RTECs during renal fibrosis is limited. In this study, we used multi‐omics to show that PC knockout can promote glycolysis through the SQOR/cGAS/STING pathways in RTECs, confirming PC as a key new molecule in the metabolic reprogramming of these cells. Therefore, PC may be a promising target for interventions in metabolic reprogramming and renal fibrosis.

## 4 Experimental Section

### Data Collection

The RNA‐seq datasets of GSE217650^[^ ^44^ ^]^ and GSE79443^[^ ^45^ ^]^ and microarray dataset GSE214358^[^ ^46^ ^]^ were obtained from the Gene Expression Omnibus (GEO) database. R (version 4.0.4) and RStudio (version 1.2.5033) were used to analyze the data in this study. scRNA‐seq data from the GSE140023 dataset were obtained from the GEO database.^[^ ^47^, ^48^ ^]^ The R package “Seurat” (version 4.0.2) was used to process the data.

### Human Kidney Samples

All human renal biopsy samples were obtained from the Department of Pathology, Xiangya Hospital, Central South University between 2019 and 2023. Renal biopsy samples from patients with DN(*n* = 10), hypertensive kidney damage (HN, *n* = 5), IgAN(*n* = 10), and ON(*n* = 3) were analyzed. In addition, five specimens obtained from total nephrectomy procedures for urological indications served as controls. Human samples were collected in accordance with the Ethics Committee of Xiangya Hospital, Central South University (approval number: 202103096). Clinical data of the patients are listed in Table S1, Supporting Information.

### Animal Studies

Male C57BL/6 mice aged 8–10 weeks and weighing 20–22 g were purchased from Hunan SJA Laboratory Animal Co., Ltd. All animal studies were approved by the Ethics Review Committee for Animal Experimentation of Central South University (approval number: CSU‐2022‐0001‐0166) and the Ethics Committee of Xiangya Hospital, Central South University (approval number: 202103096) and were performed in accordance with the NIH Guide for the Care and Use of Laboratory Animals.

*Pcx* ^flox/flox^ (Control) mice and *Pcx* ^flox/flox^ *;Cdh16‐Cre* (*Pcx* ^cKO^) mice with a C57BL/6J background were purchased from GemPharmatech LLC (Nanjing, China). To generate renal proximal tubule–specific *Pcx* gene‐knockout mice, *Pcx* ^flox/flox^ mice were crossed with *Cdh16‐Cre* mice, and the resulting *Pcx* ^flox/wt^ *;Cdh16‐Cre* mice were further crossed with *Pcx* ^flox/wt^. Only *Pcx* ^flox/flox^ and *Pcx* ^flox/flox^ *;Cdh16‐Cre* mice were used for subsequent experiments.

To generate renal proximal tubule–specific *Pcx* knockin mice, CRISPR/Cas9 technology was used to insert *Pcx* gene fragments into the H11 sites of mice (purchased from GemPharmatech LLC [Nanjing, China]). Briefly, sgRNA was transcribed in vitro; a donor vector was constructed; Cas9, donor, and sgRNA were microinjected into the fertilized eggs of C57BL/6J mice; and F0 generation mice were obtained. F0 generation‐positive mice were mated with C57BL/6J mice to obtain an F1 generation‐positive mouse model (*Pcx* ^flox/flox^, Control) with stable heredity. *Pcx* ^flox/flox^ (control) mice were crossed with *Cdh16‐Cre* mice to obtain *Pcx* ^flox/flox^ *;Cdh16‐Cre* mice (*Pcx* ^Tg^ *)*.

To observe whether the tubule‐specific knockout mice had spontaneous kidney disease, mice were sacrificed at 2 (*n* = 3) and 16 (*n* = 5) months, and the kidneys were collected for pathological, immunohistochemical, and western blot evaluations, among other tests. Renal fibrosis induced by UUO was established, as described previously.^[^ ^49^ ^]^ In short, 6–8‐weeks‐old mice were anesthetized with isoflurane, after which the left ureter was isolated and ligated. The mice were euthanized after 14 days, and the obstructed kidneys were harvested for analysis. Furthermore, another CKD mouse model was established, namely, the adenine mouse model. Mice were fed a 0.2% adenine diet or normal diet for 3 weeks and euthanized after 21 days.

### Cell Culture

Human kidney tubular epithelial cells (HK‐2) were purchased from Procell Life Science & Technology Co., Ltd., and cultured in F12/DMEM (Gibco, USA) containing 10% FBS (BioChannel, Nanjing, China) and 1% 10 000 U mL^−1^ penicillin‐streptomycin (Gibco, USA) at 37 °C in 5% CO_2_. PC knockout (PCKO) HK‐2 cells were generated using the CRISPR‐Cas9 system (Hanheng Biological Technology Co. Ltd., Shanghai, China). HK‐2 cells were exposed to a complete culture medium supplemented with 15 ng mL^−1^ TGF‐β1 (100‐21, PeproTech, USA) for 24 h. For activation of ferroptosis, Erastin (final concentration 10  µm, S7242, Selleck, USA) was added into the culture medium for 24 h. To monitor the glycolysis on fibrosis, TEPP‐46 (final concentration 20  µm, HY‐18657, MedChemExpress, USA), an activator of PKM2 was added into the culture medium for 24 h.

### Cell Transfection

The siRNAs for PC and SQOR were designed and synthesized by Genepharma (Suzhou, China). HK‐2 cells were plated onto a cell culture plate at 50–70% confluence; and then, transfected 48 h later using Lipofectamine 2000 (Invitrogen Biotechnology, USA). SiRNA was transfected at a working concentration according to the manufacturer's protocol. The sequence was as follows: PC siRNA: CCUGGUGGCCUGUACCAAATT; SQOR siRNA: CCCAGUGAGAGACAUUUCUTT.

### Histology

Kidney tissues were fixed in 4% paraformaldehyde solution for 24 h and embedded in paraffin. Paraffin sections (3 µm) were stained with hematoxylin and eosin (HE) and Masson's trichrome, and the histopathological renal damage was assessed, as described previously.^[^ ^50^ ^]^

### Immunohistochemistry and Immunofluorescence Staining

Immunohistochemistry was performed, as described previously.^[^ ^51^, ^52^ ^]^ According to the manufacturer's instructions, paraffin sections were dewaxed, dehydrated; and then, repaired with citric buffer. After natural cooling, 5% goat serum solution was added, and the section was soaked for 1 h for sealing, and then, incubated with primary antibodies (PC and collagen I) overnight at 4 °C. The following day, the primary antibody was washed, and the section was incubated with the corresponding secondary antibodies. After washing with PBS, DAB (ZSGB‐BIO, ZLI‐9018) was used to visualize the indicated proteins, followed by counterstaining with hematoxylin to identify cellular nuclei. For immunofluorescence staining, deparaffinized sections were blocked with 5% normal goat serum for 1 h; and then, incubated with primary antibodies (PC, Proteintech; LTL, vector laboratories) overnight at 4 °C, followed by staining with FITC or tetramethylrhodamine‐conjugated secondary antibodies. Sections were viewed and imaged using a Nikon N2‐DM4B fluorescence microscope.

### Transmission Electron Microscopy

For transmission electron microscopy, samples were prepared as previously described with modifications.^[^ ^53^, ^54^ ^]^ The cell samples were placed in a fixative solution (pH = 7.2; a mixture containing 2% paraformaldehyde and 2.5% glutaraldehyde) for ≈1 h. The sample was washed three times with 0.1 m PBS (pH = 7.2–7.4) buffer for 10 min each time. After fixing with a 1% osmic acid solution for 1 h, the mixture was rinsed three times with purified water for 15 min each time. The sample was dehydrated with gradient alcohol concentrations of 30%, 50%, 70%, 95%, and 100%. The dried sample was ticked to the sample stage using a double‐sided carbon tape. After completion, the metal coatings were observed and photographed using an electron microscope.

### Western Blot Analysis

Total protein was isolated from the kidneys or cells, as previously described.^[^ ^54^, ^55^ ^]^ Briefly, cultured cell or kidney tissue lysates were extracted in sodium dodecyl sulfate (SDS) buffer supplemented with a protease inhibitor cocktail (P8340, Sigma–Aldrich, USA). The protein concentration was measured using a BCA protein assay kit (23225, Thermo Fisher Scientific, USA). Equal amounts of protein were loaded in each lane, separated by SDS‐PAGE under reducing conditions, and transferred onto a PVDF membrane for the standard procedure of immunoblot analysis. Primary antibodies for immunoblot analysis included rabbit anti‐PC (16588‐1‐AP, Proteintech, USA), rabbit anti‐Fibronectin (15613‐1‐AP, Proteintech, USA), rabbit anti‐COL1A1 (R10022, Zen‐bio, Chengdu, China), rabbit anti‐vimentin (10366‐1‐AP, Proteintech, USA), mouse anti‐α‐SMA (A5228, Sigma–Aldrich, USA), rabbit anti‐P‐SMAD3 (9520S, Cell Signaling Technology, USA), rabbit anti‐Snail (3879S, Cell Signaling Technology, USA), rabbit anti‐FIS1 (ab184554, Abcam, USA), rabbit anti‐SQOR (P02738, Hunan ProMab Biotechnologies, China), rabbit anti‐MNF2 (ab205236, Abcam, China), mouse anti‐Alpha Tubulin (66031‐1‐Ig, Proteintech, USA), and mouse anti‐ACTB/β‐actin (3700S, Cell Signaling Technology, China). The secondary antibodies used for immunoblot analysis were obtained from Jackson ImmunoResearch. Antigens on the blots were revealed with a Clarity Western ECL substrate kit (BL530B, Biosharp, China; 34580, ThermoFisher Scientific, USA).

### Co‐Immunoprecipitation (CO‐IP)

The method of CO‐IP was carried out according to the manufacturer's instructions (Selleck, USA), and the specific steps were as follows: 293T cells transfected with His‐PC (ordered from Hunan Fenghui Biotechnology Co., Ltd., China) and Flag‐SQOR plasmids (ordered from Shanghai Genechem Co., Ltd., China) or HK‐2 cells after discarding the medium and washing twice with 1 × PBS; IP lysate buffer (Thermo Fisher Scientific, USA) was added and collected into 1.5 mL EP tube. The supernatant was collected by centrifugation (4 °C, 14 000 × *g*, 10 min). Target antibody and control IgG antibody were added and rotated overnight at 4 °C. On the next day, a certain amount of Protein A/G magnetic beads was taken and washed five times with washing buffer. The antigen–antibody mixture was added into the Protein A/G magnetic beads and rotated at room temperature for 1 h. Adsorption was performed with a magnetic rack to remove the remaining protein followed by four‐time washing. Finally, an appropriate amount of IP lysate buffer was added and heated at 95 °C for 5 min, and the supernatant was collected after adsorption with a magnetic frame for western blot experiments.

### Immunofluorescent Confocal Microscopy

The immunofluorescence study was performed as described previously.^[^ ^52^ ^]^ Briefly, HK‐2 cells were cultured on coverslips (Jet Biofil, Guangzhou, China), and after removing the supernatant, the cells were washed once with 1× PBS. The cells were then fixed with pre‐chilled ice‐cold methanol for 20 min and washed twice with 1× PBS. Permeabilization was performed using 0.15% Triton X‐100 in 1× PBS for 5 min, followed by two washes with 1× PBS. Blocking was carried out with 5% goat serum in 1× PBS at room temperature for 1 h. Primary antibodies, diluted in 5% goat serum in 1× PBS, were added to the cells, incubated at 37 °C for 1 h; and then, transferred to 4 °C for overnight incubation. The following day, the cells were washed three times with 1× PBS, and fluorescent secondary antibodies (Alexa Fluor 488 or Alexa Fluor 568, ordered from Thermo Fisher Scientific, USA) were applied, with incubation at 37 °C for 2 h in the dark. After three additional PBS washes, the cells were mounted using an anti‐quenching mounting medium (Solarbio, Beijing, China). Fluorescent images were acquired using a Zeiss Airyscan confocal microscope.

### In Vitro Pull‐Down Assay

His‐tagged PC was constructed into pET‐28a and expressed in BL21 *Escherichia coli*. The in vitro expression of PC protein was extracted using Ni‐TED Purose 6 Fast Flow according to the manufacturer's instructions (A42302‐05, Qianchun Bio., China). The glutathione *S*‐transferase (GST)‐tagged SQOR was constructed into pGE‐6P‐1 and expressed in BL21 *E. coil*. The purification of SQOR protein was extracted using the GST‐tag Protein Purification Kit according to the manufacturer's instructions (P2260S, Beyotime, Shanghai, China). For the pull down assay, the GST or GST‐SQOR fusion proteins were incubated with His‐PC protein in His‐tag Protein Purification (M2300, Solarbio, Beijing, China) overnight at 4 °C. The bound proteins were eluted and visualized with immunoblotting.

### Liquid Chromatography‐Tandem Mass Spectrometry (LC‒MS/MS) Analysis

Samples were sent to Jingjie PTM BioLab (Hangzhou, China) Co. Ltd., and the relative contents of various proteins were detected with a LC‐MS/MS platform. First, protein samples were subjected to in‐gel tryptic digestion, where gel pieces were sequentially destained, dehydrated, reduced with 10 mm dithiothreitol, alkylated with 55 mm iodoacetamide, and digested with trypsin at 37 °C overnight. Peptides were extracted using 50% acetonitrile/5% formic acid followed by 100% acetonitrile, dried, and resuspended in 2% acetonitrile/0.1% formic acid.

The tryptic peptides were analyzed using a home‐made reversed‐phase analytical column coupled to an EASY‐nLC 1000 UPLC system. Peptides were separated with a gradient of solvent B (0.1% formic acid in 98% acetonitrile) and analyzed using a Q Exactive Plus mass spectrometer. The *m*/*z* scan range was 350–1800 for full scanning with a resolution of 70 000, and tandem mass spectra were acquired using data‐dependent acquisition with 20 MS/MS scans per cycle at a resolution of 17 500.

The MS/MS data were processed using Proteome Discoverer 1.3 and searched against the database. Trypsin/P was specified as the cleavage enzyme with up to two missed cleavages allowed. Carbamidomethylation of Cys was set as a fixed modification, and oxidation of Met was set as a variable modification. Precursor and fragment mass tolerances were set to 10 ppm and 0.02 Da, respectively. Peptide confidence was set to high, with peptide ion scores > 20.

### RNA Isolation and Real‐Time PCR Analysis

Mouse kidney tissue and cell RNA were extracted using an RNA purification kit (12183025, TransGen Biotech, Beijing, China). Next, 1 µg of total RNA was reverse transcribed using the Reverse Transcriptase synthesis cDNA kit (K1621, Invitrogen, USA), according to the manufacturer's instructions. The Maxima SYBR Green/ROX qPCR kit (K0221, Invitrogen, USA) was used according to the manufacturer's instructions. The primer sequences used for RT‐qPCR are listed in Table S2, Supporting Information.

### ATP Detection

Cellular ATP levels were measured, according to the manufacturer's instructions (S0027, Beyotime Biotechnology, China). The cell culture medium was removed, and 200 µL of lysate was added to each well for cell lysis. The wells were centrifuged at 4 °C at 12 000 × *g* for 5 min, and the supernatant was obtained for subsequent determination. The ATP standard solution was diluted to the appropriate concentration gradients (0.01, 0.03, 0.1, 0.3, 1, 3, and 10 µm) using the ATP‐detected lysate. Next, 100 µL of ATP test solution was added to each test hole, which was left at room temperature for 3–5 min to consume all background ATP. Next, a 20 µL sample or standard was added to each test hole and quickly mixed using a micropipette. The RLU or CPM values were measured for at least 2 s using a chemiluminescent or liquid flash meter. A standard curve was established to calculate the ATP content.

### Reactive Oxygen Species (ROS) Detection

MitoSOX Red powder (M36008, Thermo Scientific, USA) was dissolved in dimethyl sulfoxide (DMSO) to a storage solution concentration of 5 mm, and the storage solution was diluted with Hank's balanced salt solution (HBSS) buffer to a working solution concentration of 500 nm before dyeing. Working droplets were added to the cell culture wells, which were incubated at 37 °C and 5% carbon dioxide for 30 min. The cells were then gently washed with HBSS buffer, observed, and photographed using a fluorescence microscope (N2‐DMi8, Leica, Germany).

### JC‐1 Assay

A mitochondrial membrane potential detection kit (JC‐1) from the Beijing Solarbio Company (M8650) was used for the experiments. First, a JC‐1 dye solution was prepared. An appropriate amount of 200 × JC‐1 was diluted with ultrapure water and stirred thoroughly for dissolution and mixing. Next, 5 × JC‐1 dyeing buffer was added and mixed well to obtain the JC‐1 dyeing working solution. After discarding the cell medium, 1 mL of new cell culture medium was added. Next, 1 mL of JC‐1 dye solution was added and mixed thoroughly. The samples were incubated in a cell incubator at 37 °C for 20 min. During incubation, a sufficient amount of 1 × JC‐1 staining buffer was prepared and placed in an ice bath. After incubation, the supernatant was removed, and the cells were washed twice with 1 × JC‐1 staining buffer. Next, 2 mL of cell culture solution was added. Finally, a molecular device (Invitrogen, USA) was used for detection. The excitation and emission wavelengths were set to 490 and 530 nm, respectively, to detect the JC‐1 monomer, and 525 and 590 nm, respectively, to detect the JC‐1 polymer.

### Seahorse Assay

The OCR and ECAR were measured to determine mitochondrial function and glycolysis ability. According to the XF Cell Mito Stress Test Kit (103015‐100, Agilent, USA) and XF Glycolysis Stress Test Kit (103020‐100, Agilent, USA), HK‐2 cells were cultured with TGF‐β1 for 24 h and washed twice with the configured detection buffer (containing glucose, glutamine, and pyruvate, which was not required for the glycolysis stress test). After adding the different reagents, a Seahorse XF 96 instrument was used for detection. Data were processed using Wave 2.6.3.

### RNA‐Seq and Enrichment Analysis

Total RNA was extracted from the samples and sent to Beijing Berry Biotechnology Co., Ltd. An Illumina NovaSeq 6000 platform was used for RNA‐Seq transcriptome sequencing. After the original data were filtered and compared with the reference genome, the counts and FPKM values of the corresponding genes were obtained. Log_2_|FC| > 1.0 and adjusted‐*p* < 0.05 were used as the standards for differential gene analysis and screening of differential genes.

The “clusterProfiler” package (version 4.6.2; http://www.bioconductor.org/packages/release/bioc/html/clusterProfiler.html) of R language (version 4.2.2) for enrichment analysis of GO and Kyoto Encyclopedia of Genes and Genomes (KEGG) was used. GO analysis revealed three main processes: biological processes, molecular functions, and cellular components. Statistical significance was set at *p* < 0.05.

GSEA and gene set variation analysis (GSVA) were used to explore the underlying molecular mechanisms of the prognostic model. Enrichment terms related to Reactome, KEGG, hallmark, NABA, GO, and WikiPathways in MSigDB^[^ ^56^ ^]^ were screened using GSEA and GSVA. Volcano plots were generated using the Enhanced Volcano package (version 1.18.0). Statistical significance was set at *p* < 0.05.

### Metabolomics Analysis

Cells were collected and sent to Wuhan Metville Biological Technology Co., Ltd after quick freezing. Sixty‐eight types of energy metabolites were detected using a LC‐MS/MS platform. After quality control, the original data after disembarkation were analyzed for differential metabolites. The variable importance in projection (VIP) was obtained using the OPLS‐DA model. Log_2_|FC| > 1.0 and VIP > 1.0 were used as the standards for differential metabolite analysis and screening of differential metabolites.

### In Vivo Ubiquitination Assays

PC, SQOR, and HA‐Ub plasmids (ordered from Shanghai Genechem Co., Ltd., China) were transfected into 293T cells for 48 h. All cells were treated with MG132 (catalog HY‐13259, MedChemExpress, USA) (10 µm) for 6 h before collection. Ubiquitination of SQOR was performed with an IP assay using an anti‐SQOR antibody followed by western blot analysis with an anti‐HA antibody. To inhibit the degradation of the SQOR protein, autophagy inhibitor chloroquine (CQ, S6999, Selleck, USA) and proteasome inhibitor MG132 were used in HK‐2 cells.

### Analysis of the Protein Half‐Life

Control or PC knockdown HK‐2 cells were treated with cycloheximide (catalog S7418, Selleck, USA) or DMSO for the indicated durations (0, 1, 3, and 6 h). After lysis, western blot analysis was carried out using an anti‐SQOR antibody.

### Statistical Analysis

Data were pre‐processed to ensure quality and consistency, including normalization and evaluation of outliers. Results are presented as mean ± SEM, with the sample size (*n*) specified in the figure legends for each experiment.

For comparisons between two groups, an unpaired two‐tailed *t*‐test was used. For comparisons involving multiple groups, one‐way ANOVA was performed, followed by Tukey's post hoc test to assess significant differences. The correlation between the two variables was measured by Pearson's correlation coefficient (*r*). Statistical assumptions, including normality and homogeneity of variance, were validated prior to analysis. A *p‐*value of < 0.05 was considered statistically significant. A single asterisk was used for *p*‐values < 0.05, two asterisks for *p* < 0.01, and three asterisks for *p* < 0.001.

Software and tools used for statistical analyses included GraphPad Prism 10 for graphical and statistical assessments and SPSS 20 for data management.

## Conflict of Interest

The authors declare no conflict of interest.

## Author Contributions

H.H. and Y.H. conducted in vivo and in vitro experiments, performed data analysis, and drafted the manuscript. Y.Z., J.Z., X.H., J.C., S.W., and Y.X. participated in the animal experiments. Y.X., Q.Y., L.H., J.M., and L.T. contributed to the experimental design. H.Y. analyzed human renal biopsy samples. Z.P. designed this experiment and revised the manuscript.

## Supporting information



Supporting Information

## Data Availability

All data generated or analysed during this study are included in this published article.
