# Supplementary material for: Deletion of Pyruvate Carboxylase in Tubular Epithelial Cell Promotes Renal Fibrosis by Regulating SQOR/cGAS/STING‐Mediated Glycolysis
Source: Adv Sci (Weinh). 2025 Jan 21;12(13):2408753. doi: 10.1002/advs.202408753 (PMC11967762; doi:10.1002/advs.202408753)
Supplement: Supplementary file 1 — Supporting Information [file ADVS-12-2408753-s001.docx]

Supplementary Figures and Tables

# Supplementary Figures


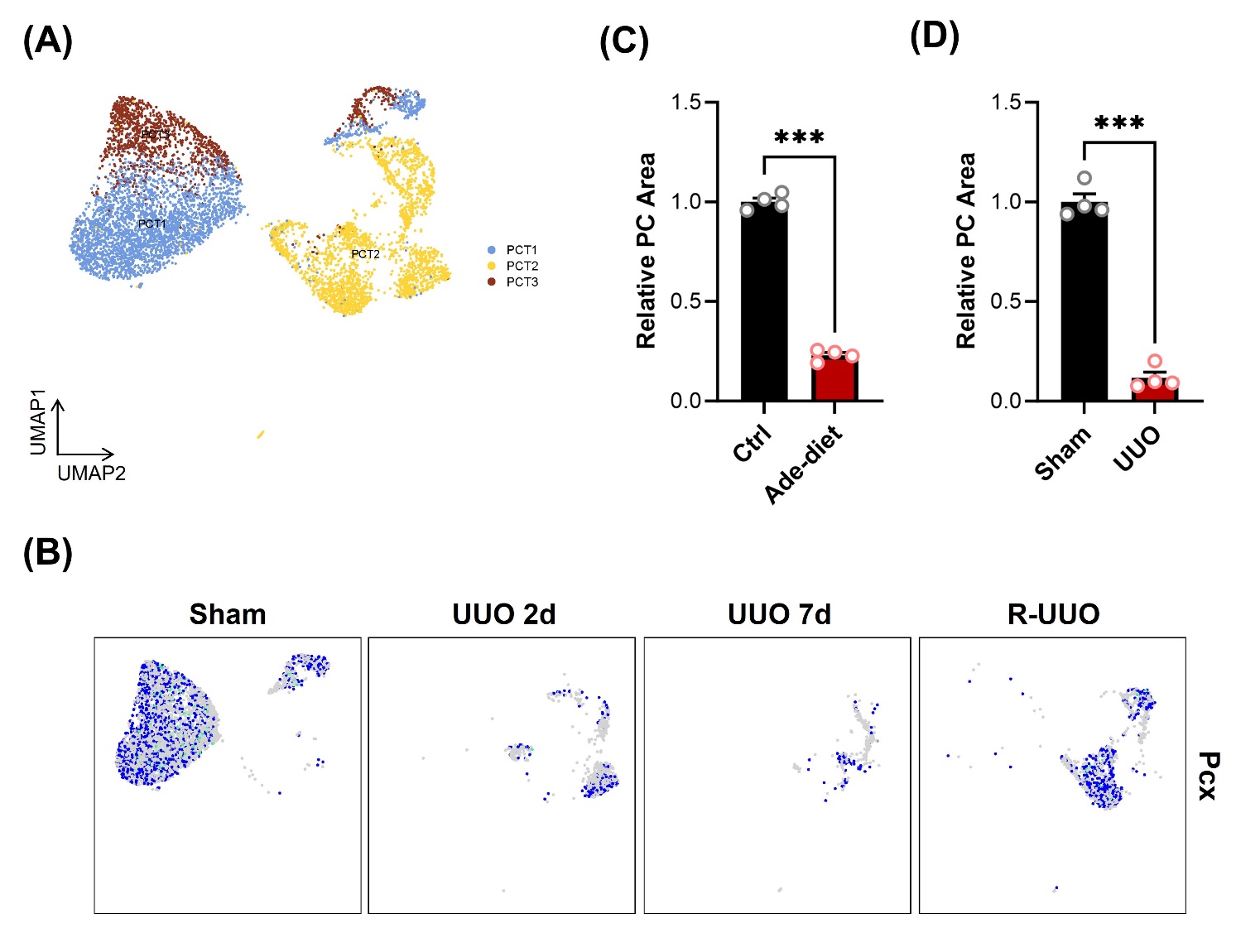


**Figure S1.** **(A)** Composition and distribution of single proximal convoluted tubule (PCT) cells from GSE140023. **(B)** Uniform manifold approximation and projection (UMAP) of GSE140023 dataset showing *Pcx* expression pattern in mouse PCT cells that underwent sham or unilateral ureteral obstruction (UUO) operations. **(C)** Statistical graph for immunofluorescence for PC/LTL in control kidneys and those of mice subjected to 21 days of adenine diet in **Figure 1I**. **(D)** Statistical graph for immunofluorescence for PC/LTL in sham kidneys and those of mice subjected to 14 days of unilateral ureteral obstruction (UUO) in **Figure 1J**. Results are expressed as mean ± SEM. ***P < 0.001.


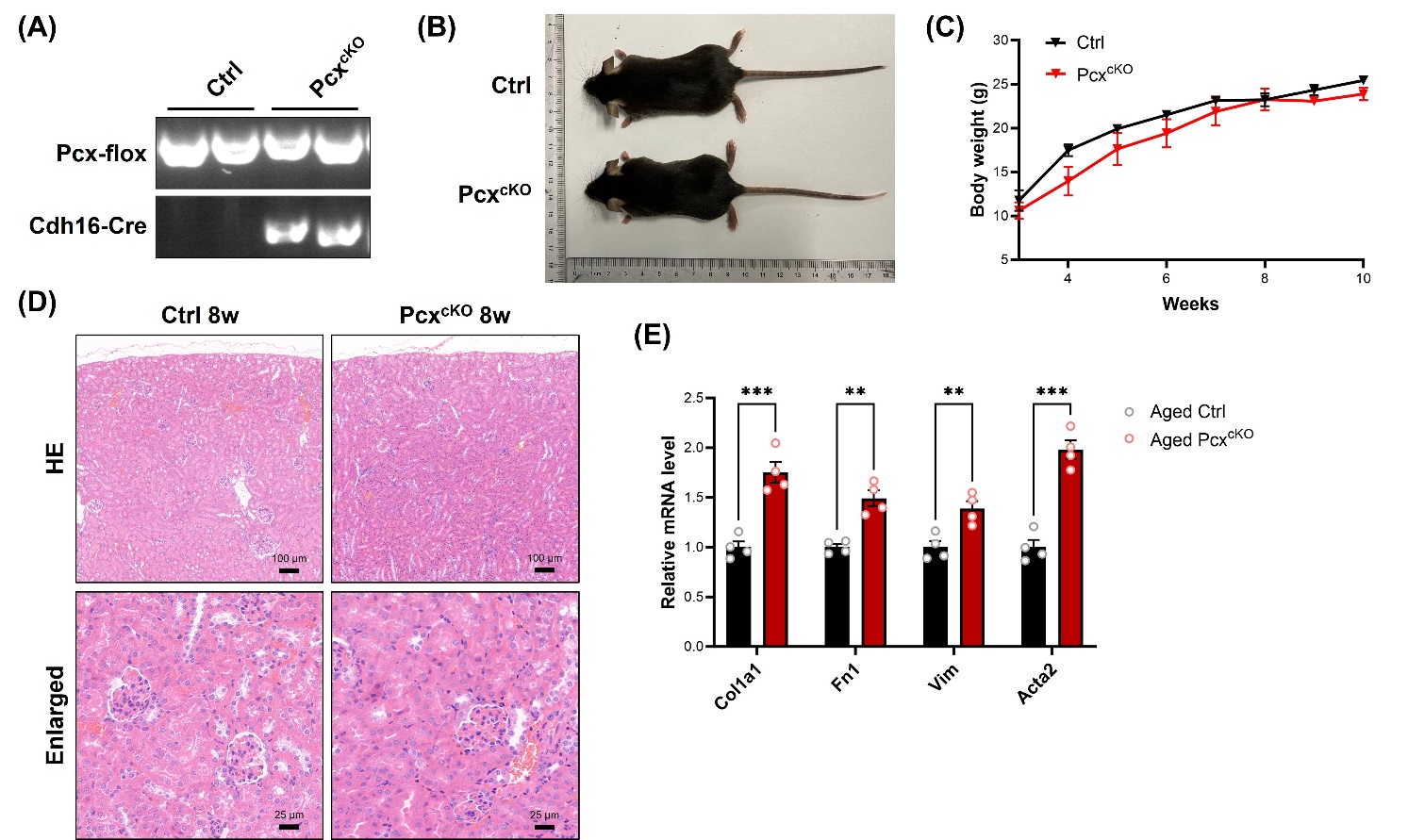


**Figure S2.** **(A)** Identification of mouse genotypes by agarose gel electrophoresis. **(B, C)** Body shape and weight of control mice (*Pcx^flox/flox^*, n = 7) and *Pcx^flox/flox^;Ksp-Cre* mice (*Pcx^cKO^*, n = 7). **(D)** Representative images and statistical graphs for HE staining in renal tissues from control and *Pcx^cKO^* mice aged 2-months-old. **(E)** Real-time polymerase chain reaction (RT-PCR) revealed the mRNA levels of *Col1a1*, *Fn1*, *Vim*, and *Acta2* in control and *Pcx^cKO^* mice aged 16-months-old. Scale bar, 100 μm; enlarged, 25 μm. Results are expressed as mean ± SEM. **P < 0.01; ***P < 0.001.


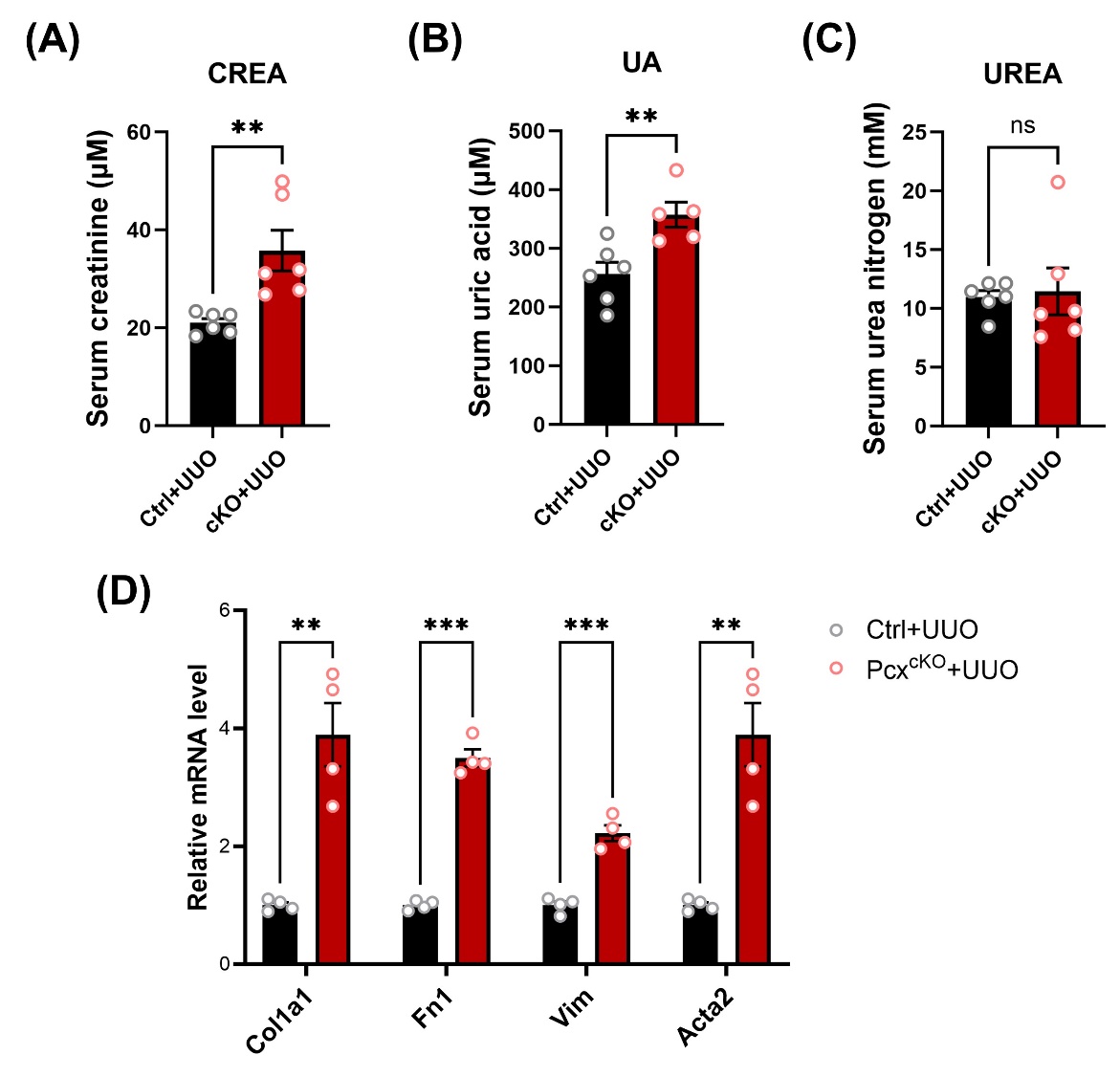


**Figure S3.** **(A)** Serum creatinine, **(B)** uric acid, and **(C)** urea nitrogen from the indicated animal groups were determined and quantified. **(D)** Real-time polymerase chain reaction (RT-PCR) revealed the mRNA levels of *Col1a1*, *Fn1*, *Vim*, and *Acta2* in control and *Pcx^cKO^* mice subjected to 14 days of unilateral ureteral obstruction (UUO). Results are expressed as mean ± SEM. **P < 0.01; ***P < 0.001; ns, not significant.


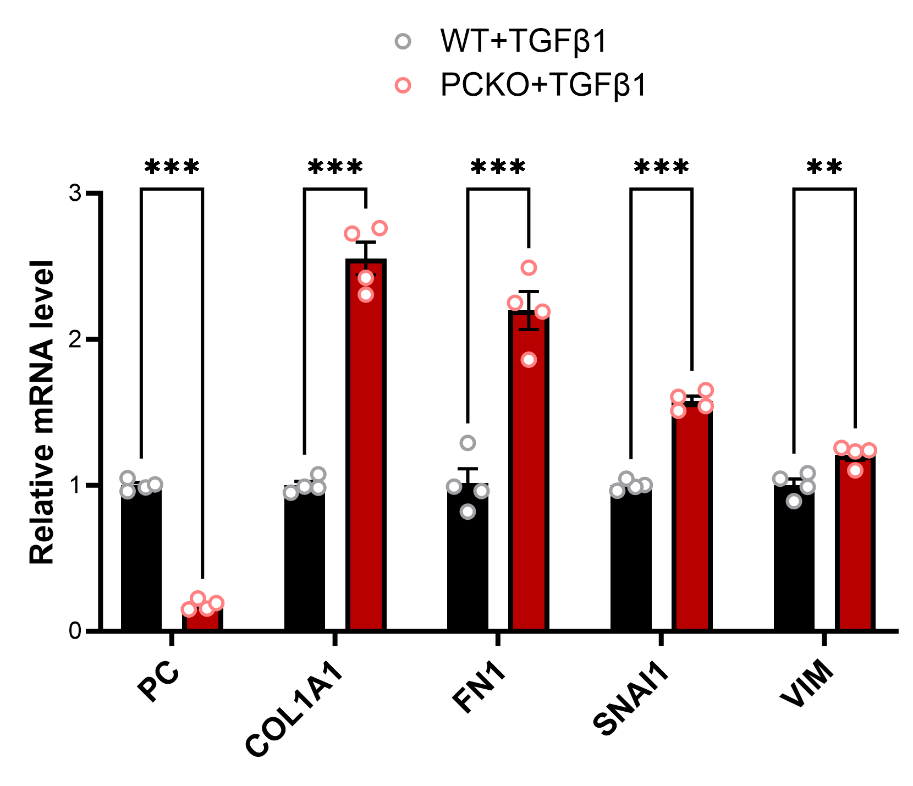


**Figure S4.** Real-time polymerase chain reaction (RT-PCR) revealed the mRNA levels of *PC*, *COL1A1*, *FN1, SNAI1*, and *VIM* in HK-2 cells underwent stable knock-out of control or PC (WT or PCKO) genes and were stimulated with TGF-β1 for 24 h (15 ng/mL). Results are expressed as mean ± SEM. **P < 0.01; ***P < 0.001.


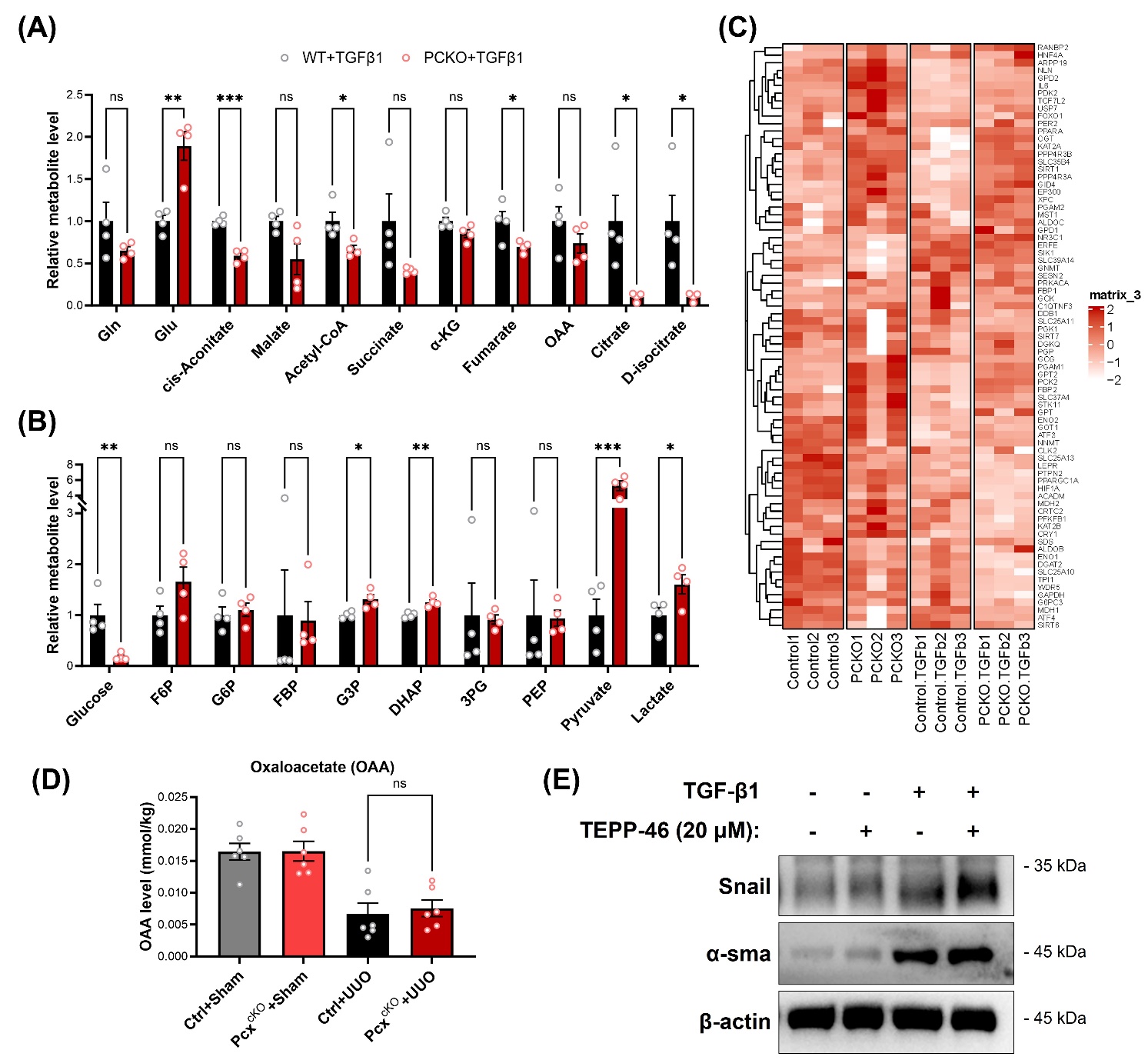


**Figure S5.** Relative levels of **(A)** oxidative phosphorylation-associated and **(B)** glycolysis-associated metabolites in the targeted metabolomics data. **(C)** The gluconeogenesis genes expression patten on the RNA-Seq landscape of the PCKO+TGF-β1 cells compared to WT+TGF-β1 cells. **(D)** The oxaloacetate (OAA) content in kidney tissues from control and *Pcx^cKO^* mice subjected to sham operation or 14 days after the UUO operation. **(E)** Western blot analysis of fibrosis markers Snail and α-sma expression in HK-2 cells treated with glycolytic activator TEPP-46 and TGF-β1. Results are expressed as mean ± SEM. *P < 0.05; **P < 0.01; ***P < 0.001; ns, not significant.


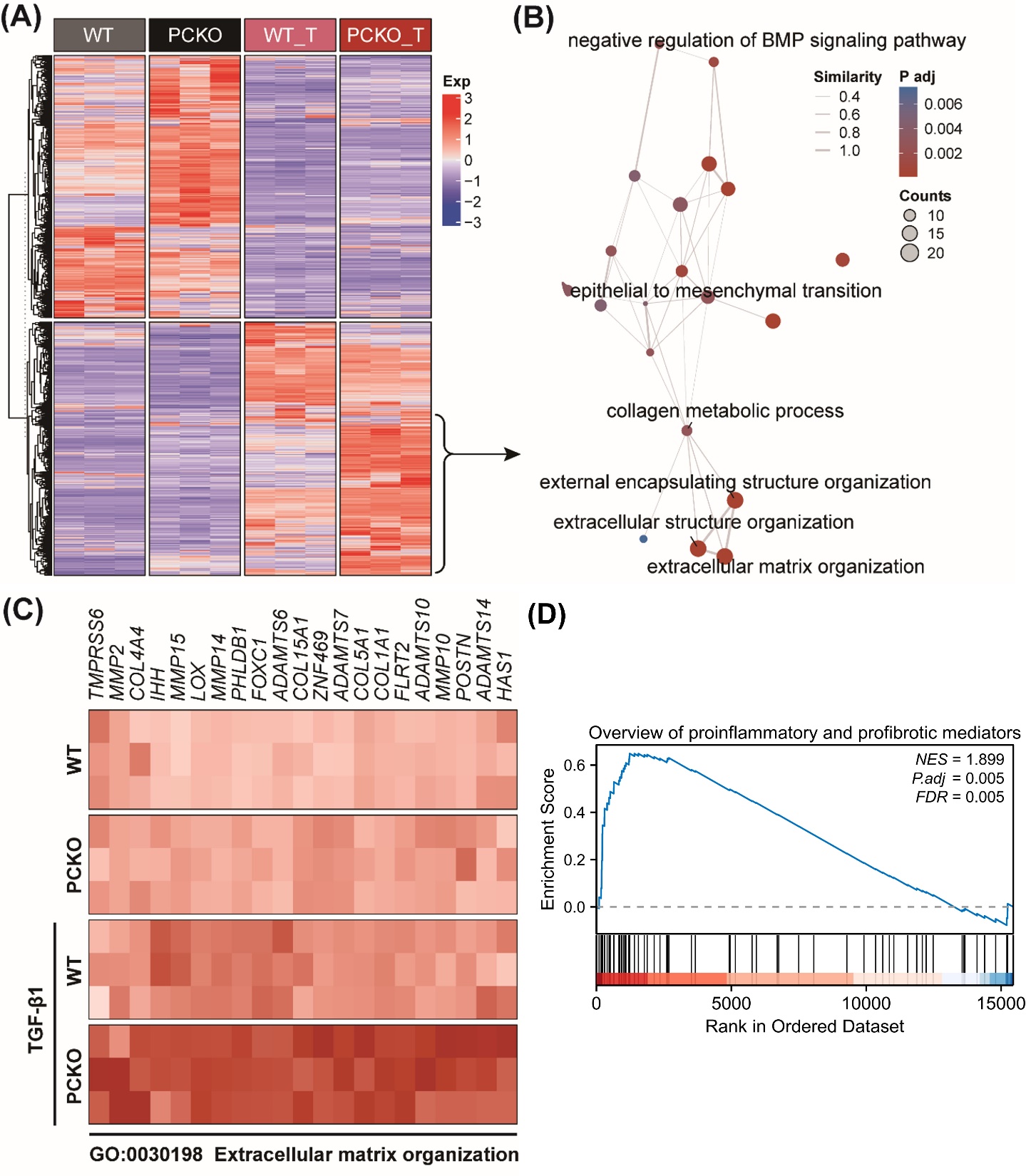


**Figure S6.** **(A)** Heatmap of the differentially expressed genes from RNA-Seq data of HK-2 cells. The color in heatmaps from blue to red shows the progression from low expression to high expression, respectively. **(B)** Emapplot of the GO biological process analyses performed on selected genes from the heatmap. **(C)** Heatmap of genes in GO term “GO:0030198 Extracellular matrix organization”. **(D)** The gene set enrichment analysis (GSEA) for the term of “overview of proinflammatory and profibrotic mediators” on the RNA-Seq landscape of the PCKO+TGF-β1 cells compared to WT+TGF-β1 cells.


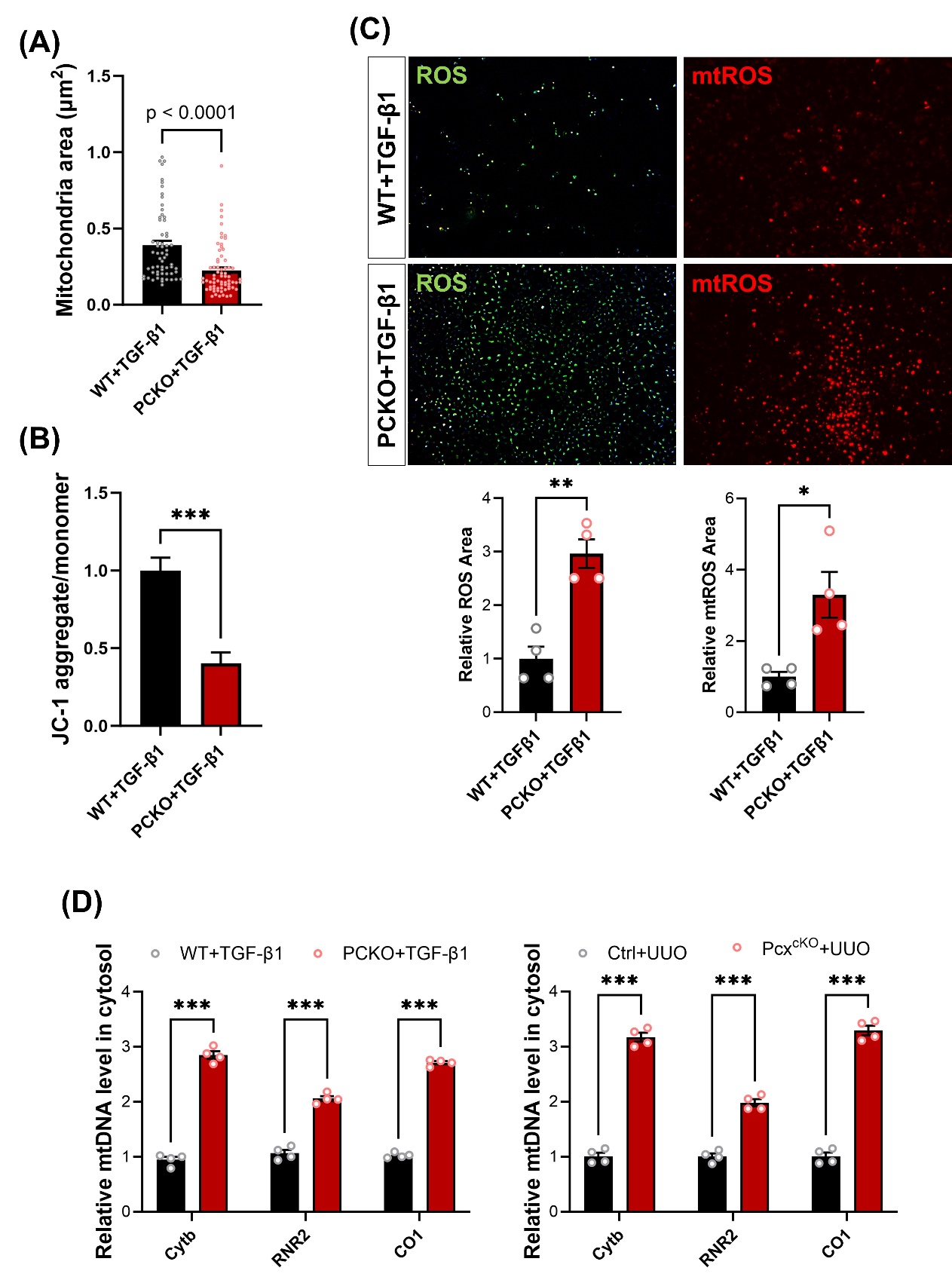


**Figure S7.** **(A)** Statistical graphs of mitochondria from electron micrograph in each group. **(B)** The ratio of JC-1 aggregated to JC-1 monomers in WT+TGF-β1 and PCKO+TGF-β1 groups. **(C)** Representative images of intracellular ROS and mitochondrial ROS under fluorescence microscope of HK-2 cell of WT+TGF-β1 and PCKO+TGF-β1 groups. **(D)** Real-time polymerase chain reaction (RT-PCR) revealed the mRNA levels of mtDNA (CytB, RNR2, and CO1) in WT+TGF-β1 and PCKO+TGF-β1 groups, as well as *Pcx^flox/flox^*+UUO and *Pcx^cKO^*+UUO groups. Results are expressed as mean ± SEM. *P < 0.05; **P < 0.01; ***P < 0.001.


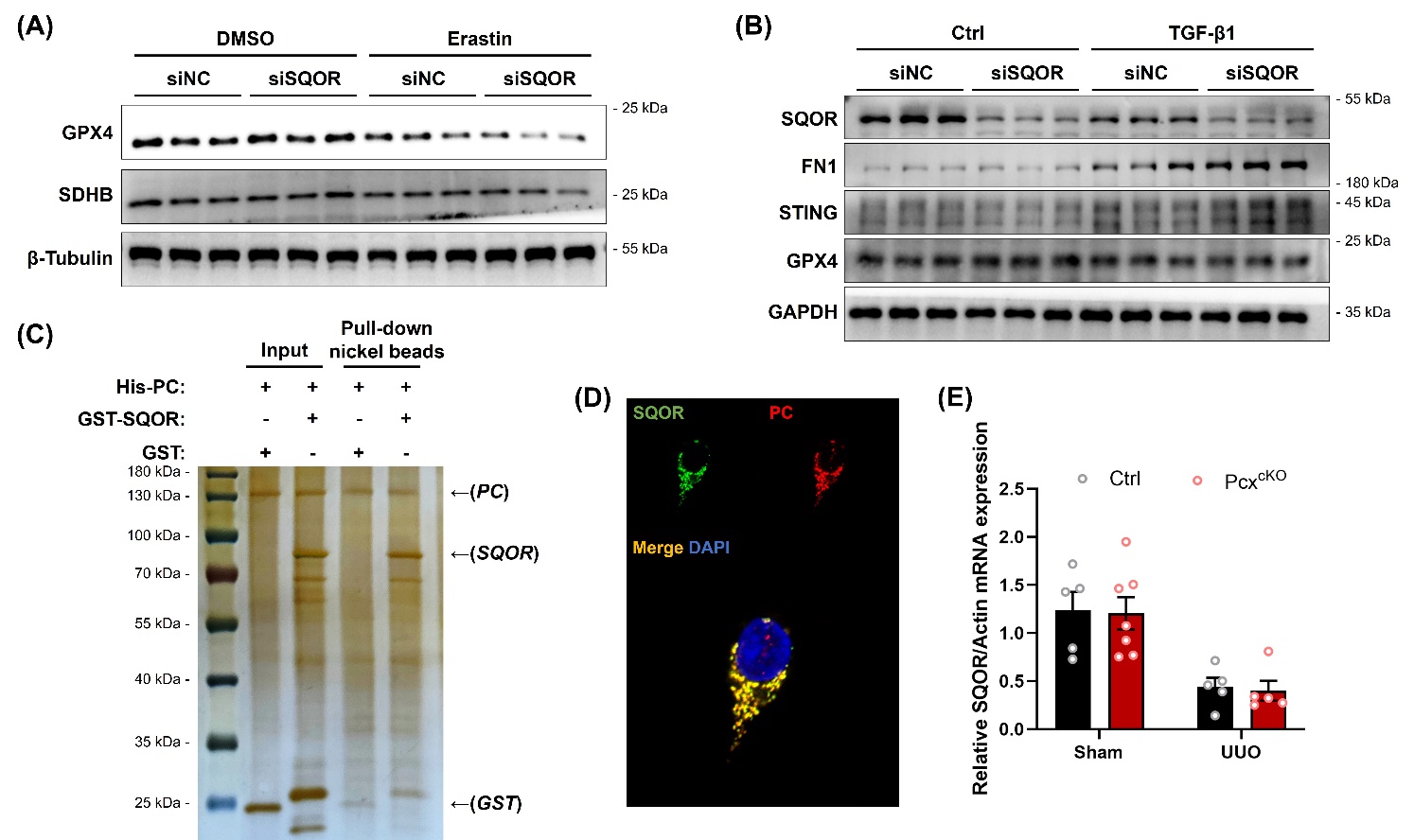


**Figure S8.** **(A)** Western blot analysis of ferroptosis marker GPX4 and SDHB expression in HK-2 cells silencing SQOR and treated with ferroptosis inducer erastin. **(B)** Western blot analysis of SQOR, FN1, STING, and GPX4 expression in HK-2 cells silencing SQOR and treated with TGF-β1. **(C)** GST and GST-SQOR were purified from *E. coli* and analyzed by gel silver staining**. (D)** Confocal analysis of SQOR and PC in HK-2 cells. **(E)** Real-time polymerase chain reaction (RT-PCR) revealed the mRNA levels of SQOR in *Pcx^flox/flox^*+UUO and *Pcx^cKO^*+UUO groups. Results are expressed as mean ± SEM.


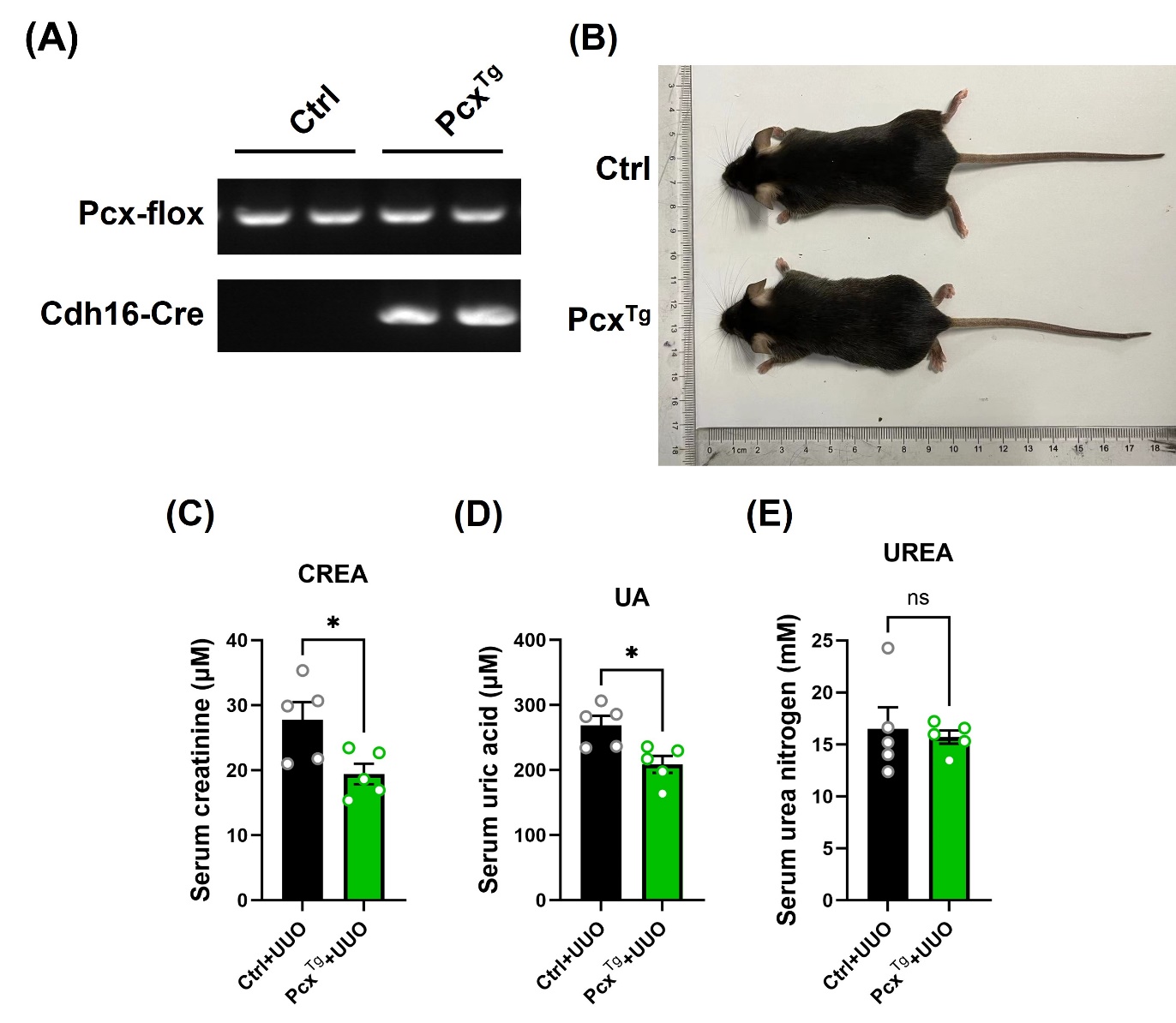


**Figure S9.** **(A)** Identification of mouse genotypes by agarose gel electrophoresis. **(B)** Body shape of control mice (*Pcx^flox/flox^*, n = 5) and tubular epithelial cell-specific *Pcx* overexpressing (*Pcx^Tg^*) mice (*Pcx^Tg^*, n = 5). **(C)** Serum creatinine, **(D)** uric acid, and **(E)** urea nitrogen from the indicated animal groups were determined and quantified. Results are expressed as mean ± SEM. *P < 0.05; ns, not significant.

# Supplementary Tables

**Table S1. Clinical information of CKD patients**

| **Patient No.** | **Gender** | **Age (year)** | **Pathological diagnosis** | **Serum creatinine (μM)** | **eGFR** | **CKD stage** |
| --- | --- | --- | --- | --- | --- | --- |
| P1 | Female | 50 | Hypertensive nephropathy | 331.7 | 13.3 | 5 |
| P2 | Male | 50 | Diabetic nephropathy | 165 | 41.1 | 3 |
| P3 | Male | 57 | Diabetic nephropathy | 494 | 10.4 | 5 |
| P4 | Female | 28 | Diabetic nephropathy | 112 | 57.5 | 3 |
| P5 | Male | 31 | IgA nephropathy | 100 | 86 | 2 |
| P6 | Female | 31 | IgA nephropathy | 143 | 41.9 | 3 |
| P7 | Male | 17 | IgA nephropathy | 56.1 | 144.5 | 1 |
| P8 | Female | 27 | IgA nephropathy | 98.2 | 67.9 | 2 |
| P9 | Female | 15 | Other | 69 | 113.2 | 1 |
| P10 | Female | 31 | IgA nephropathy | 48 | 125.5 | 1 |
| P11 | Female | 30 | IgA nephropathy | 133 | 46.1 | 3 |
| P12 | Female | 24 | IgA nephropathy | 343 | 15.3 | 4 |
| P13 | Male | 32 | Hypertensive nephropathy | 407.7 | 15.6 | 5 |
| P14 | Female | 35 | Minimal change disease | 48.8 | 121.4 | 1 |
| P15 | Male | 31 | Hypertensive nephropathy | 513.9 | 11.1 | 5 |
| P16 | Male | 41 | Hypertensive nephropathy | 74 | 108.9 | 1 |
| P17 | Female | 7.13 | Other | 26 | 174.3 | 1 |
| P18 | Male | 31 | Hypertensive nephropathy | 133.5 | 60.7 | 2 |
| P19 | Male | 52 | Diabetic nephropathy | 94 | 80 | 2 |
| P20 | Male | 49 | Diabetic nephropathy | 225 | 28.4 | 4 |
| P21 | Female | 62 | Diabetic nephropathy | 128 | 38.6 | 3 |
| P22 | Male | 43 | Diabetic nephropathy | 74 | 107.4 | 1 |
| P23 | Male | 51 | Diabetic nephropathy | 286 | 21 | 4 |
| P24 | Male | 70 | IgA nephropathy | 194 | 29.4 | 4 |
| P25 | Male | 39 | Diabetic nephropathy | 106 | 75.8 | 2 |
| P26 | Female | 33 | Diabetic nephropathy | 59 | 59.4 | 3 |
| P27 | Female | 32 | Other | 48.7 | 124 | 1 |
| P28 | Male | 42 | IgA nephropathy | 105 | 75.1 | 2 |
| P29 | Male | 42 | IgA nephropathy | 189 | 36.9 | 3 |
| P30 | Male | 39 | Hypertensive nephropathy | 282.7 | 23.1 | 4 |
| P31 | Male | 70 | Other | 431 | 11.2 | 5 |
| P32 | Male | 30 | Other | 47.1 | 141.7 | 1 |
| P33 | Female | 64 | Obstructive nephropathy | 73.9 | 73.9 | 2 |
| P34 | Female | 71 | Obstructive nephropathy | 83 | 61.1 | 2 |
| P35 | Male | 67 | Obstructive nephropathy | 114.9 | 56.5 | 3 |

CKD, chronic kidney disease; eGFR, estimated glomerular filtration rate.

**Table S2. Primer sequence**

| **Name** | **Sequence (5’→3’)** |
| --- | --- |
| qACTB_Hs_F | ACCTTCTACAATGAGCTGCG |
| qACTB_Hs_R | CCTGGATAGCAACGTACATGG |
| qVim_Hs_F | CGTGAATACCAAGACCTGCTC |
| qVim_Hs_R | GGAAAAGTTTGGAAGAGGCAG |
| qCOL1A1_Hs_F | CCCCTGGAAAGAATGGAGATG |
| qCOL1A1_Hs_R | TCCAAACCACTGAAACCTCTG |
| qFN1_Hs_F | ACTGTACATGCTTCGGTCAG |
| qFN1_Hs_R | AGTCTCTGAATCCTGGCATTG |
| qSNAI1_Hs_F | GGAAGCCTAACTACAGCGAG |
| qSNAI1_Hs_R | CAGAGTCCCAGATGAGCATTG |
| qACTA2_Hs_F | AATGCAGAAGGAGATCACGG |
| qACTA2_Hs_R | TCCTGTTTGCTGATCCACATC |
| qACTB_Ms_F | ACCGTGAAAAGATGACCCAG |
| qACTB_Ms_R | AGCCTGGATGGCTACGTACA |
| qFn1_Ms_F | CTTTGGCAGTGGTCATTTCAG |
| qFn1_Ms_R | ATTCTCCCTTTCCATTCCCG |
| qCol1a1_Ms_F | CATAAAGGGTCATCGTGGCT |
| qCol1a1_Ms_R | TTGAGTCCGTCTTTGCCAG |
| qVim_Ms_F | TTTCTCTGCCTCTGCCAAC |
| qVim_Ms_R | TCTCATTGATCACCTGTCCATC |
| Ksp-Cre_F | GCAGATCTGGCTCTCCAAAG |
| Ksp-Cre_R | AGGCAAATTTTGGTGTACGG |
| PcxTg_F | GGGCAGTCTGGTACTTCCAAGCT |
| PcxTg_R | TGGCGTTACTATGGGAACATACGTC |
| PcxcKO_5Loxp_F | GCCCATCAGTCCTCAACTCAATG |
| PcxcKO_5Loxp_R | GGGTGAGAACAGTAATCACCAAGGC |
| PcxcKO_3Loxp_F | TGCAGCTTCCTATAAGATTTGTACCAG |
| PcxcKO_3Loxp_R | CCCTGATACTGAGCAAGCTAATCTTGG |

**Table S3. Differential expressed genes of RNA transcriptome sequencing of PCKO and control HK-2 cells after TGF-β1 stimulation**

| **Genes** | **Log_2_FoldChange** | **p-value** | **Adjusted p-value** |
| --- | --- | --- | --- |
| *TSPYL5* | -6.2345 | 6.6E-282 | 1.1E-277 |
| *VGF* | -2.69317 | 1.7E-216 | 1.3E-212 |
| *GJA1* | 2.405919 | 5.3E-183 | 2.9E-179 |
| *COL8A1* | -2.62233 | 8.1E-175 | 3.3E-171 |
| *RNF182* | 4.163102 | 1.9E-158 | 6E-155 |
| *IAH1* | -4.68702 | 1.6E-149 | 4.4E-146 |
| *IFFO2* | -1.52854 | 1.9E-132 | 4.3E-129 |
| *HMOX1* | 1.64871 | 5E-128 | 1E-124 |
| *SCG2* | 4.577574 | 1.7E-117 | 3.1E-114 |
| *IGFBP3* | -1.82857 | 4.9E-110 | 7.8E-107 |
| *G0S2* | -2.17654 | 1.2E-102 | 1.7E-99 |
| *TENM4* | -2.92619 | 5.71E-97 | 7.66E-94 |
| *SYT13* | -1.73951 | 1.02E-95 | 1.27E-92 |
| *NEFH* | -1.31561 | 1.1E-94 | 1.26E-91 |
| *SP1* | -1.31128 | 1.45E-93 | 1.56E-90 |
| *FAM167A* | 3.105232 | 3.42E-92 | 3.44E-89 |
| *TSPAN2* | -1.77953 | 5.84E-92 | 5.53E-89 |
| *SLC7A11* | 1.927103 | 2.37E-88 | 2.12E-85 |
| *ANK3* | -6.12699 | 7.72E-87 | 6.54E-84 |
| *LAMB3* | -1.3763 | 1.38E-86 | 1.11E-83 |
| *IDH1* | 1.507154 | 2.79E-83 | 2.14E-80 |
| *DSC3* | -2.23669 | 2.29E-82 | 1.68E-79 |
| *EPPK1* | -2.04322 | 1.44E-81 | 1.01E-78 |
| *SLC16A2* | 1.298961 | 1.14E-80 | 7.63E-78 |
| *PPP2R2C* | 2.692317 | 7.08E-79 | 4.56E-76 |
| *PAPPA* | -1.5018 | 2.99E-78 | 1.85E-75 |
| *TCEA3* | -2.28451 | 1.9E-77 | 1.14E-74 |
| *TLL1* | -2.48786 | 1.46E-75 | 8.39E-73 |
| *TSPAN5* | 2.272367 | 1.73E-74 | 9.29E-72 |
| *CDCP1* | -1.1992 | 1.73E-74 | 9.29E-72 |
| *F11R* | -1.22929 | 4.59E-73 | 2.39E-70 |
| *ADGRL2* | -1.97314 | 2.54E-71 | 1.28E-68 |
| *FGFR1* | -1.04038 | 3.6E-65 | 1.76E-62 |
| *GPC6* | -1.59612 | 5.45E-65 | 2.58E-62 |
| *SERPINE2* | 1.199909 | 1.7E-63 | 7.83E-61 |
| *NIFK* | -1.08967 | 6.58E-63 | 2.95E-60 |
| *IFI30* | -1.27314 | 2E-62 | 8.72E-60 |
| *FUT9* | -3.00677 | 9.56E-60 | 4.05E-57 |
| *CCPG1* | 1.170257 | 1.24E-59 | 5.11E-57 |
| *PCDH1* | 1.924432 | 2.76E-59 | 1.11E-56 |
| *ULK1* | 1.623345 | 3.22E-56 | 1.27E-53 |
| *APC2* | -1.33763 | 7.32E-56 | 2.81E-53 |
| *ADAMTS9* | -1.08968 | 5.94E-54 | 2.22E-51 |
| *DTL* | -1.03807 | 1.84E-53 | 6.75E-51 |
| *UNC45A* | -1.1368 | 2.34E-53 | 8.38E-51 |
| *FAM107B* | -1.00539 | 3.21E-53 | 1.12E-50 |
| *S100A2* | -1.24461 | 6.89E-53 | 2.36E-50 |
| *LGALS3BP* | 1.13988 | 3.43E-52 | 1.15E-49 |
| *ENG* | 1.215464 | 1.21E-51 | 3.97E-49 |
| *NPNT* | -1.08335 | 4.22E-51 | 1.33E-48 |
| *SYNE1* | 1.148361 | 1.11E-50 | 3.45E-48 |
| *SPNS2* | -1.52158 | 1.38E-50 | 4.19E-48 |
| *EPHA4* | 1.286864 | 8.56E-49 | 2.51E-46 |
| *C1S* | 1.664394 | 8.81E-49 | 2.53E-46 |
| *EPCAM* | -1.56183 | 4.88E-48 | 1.35E-45 |
| *COL1A1* | 1.180745 | 2.61E-46 | 7.02E-44 |
| *BAG1* | -1.1343 | 7.5E-46 | 1.89E-43 |
| *CBLN2* | -2.20334 | 2.32E-45 | 5.66E-43 |
| *ADM* | -1.16962 | 8.96E-45 | 2.12E-42 |
| *THOC7* | -1.25925 | 4.04E-44 | 9.43E-42 |
| *PDHB* | -1.22552 | 7.27E-44 | 1.67E-41 |
| *NRK* | 2.131422 | 1.38E-43 | 3.13E-41 |
| *GPATCH4* | -1.00624 | 1.78E-43 | 3.99E-41 |
| *ANKS1B* | 2.120118 | 4.28E-43 | 9.45E-41 |
| *SORCS2* | 1.952721 | 5.92E-43 | 1.29E-40 |
| *DPP4* | -1.02582 | 2.3E-42 | 4.87E-40 |
| *GFPT2* | 2.071151 | 2.79E-42 | 5.83E-40 |
| *SH3PXD2B* | 1.255793 | 3.64E-42 | 7.52E-40 |
| *SNPH* | 1.788401 | 1.14E-41 | 2.32E-39 |
| *SPINT1* | -1.55428 | 2.14E-41 | 4.25E-39 |
| *COL4A5* | 1.893702 | 2.75E-41 | 5.39E-39 |
| *KIF2C* | -1.00551 | 3.67E-41 | 7.03E-39 |
| *ZNF618* | 1.38985 | 4.64E-41 | 8.79E-39 |
| *TCF21* | 2.875537 | 5.24E-41 | 9.81E-39 |
| *RYBP* | -1.02985 | 6.19E-41 | 1.13E-38 |
| *ZNF608* | 1.07477 | 7.21E-41 | 1.31E-38 |
| *PCK2* | 1.269871 | 1.01E-40 | 1.77E-38 |
| *STAT2* | 1.021942 | 1.94E-40 | 3.35E-38 |
| *DCLK1* | -2.19755 | 3.04E-40 | 5.21E-38 |
| *PLS1* | -1.15339 | 3.74E-40 | 6.35E-38 |
| *CHAC1* | 1.38756 | 4.37E-40 | 7.34E-38 |
| *CTSH* | -1.17104 | 4.82E-40 | 8E-38 |
| *ASNS* | 1.306684 | 3.93E-39 | 6.45E-37 |
| *PTPRG* | -1.1377 | 5.5E-39 | 8.95E-37 |
| *KCNIP1* | -1.71446 | 1.15E-38 | 1.84E-36 |
| *RASSF1* | -1.09911 | 1.61E-38 | 2.55E-36 |
| *SCARF1* | -1.00046 | 6.42E-38 | 9.63E-36 |
| *TAGLN3* | -1.6113 | 6.46E-38 | 9.63E-36 |
| *PODXL* | 1.392323 | 2.09E-37 | 3.03E-35 |
| *FBXO2* | -1.68153 | 6.32E-37 | 9.02E-35 |
| *SHQ1* | -1.15117 | 7.28E-37 | 1.03E-34 |
| *ZNF626* | -4.33165 | 8.2E-36 | 1.09E-33 |
| *C9orf84* | 1.599591 | 8.5E-36 | 1.12E-33 |
| *SPOCD1* | -1.06899 | 5.42E-35 | 7.04E-33 |
| *ID1* | 1.236948 | 4.08E-34 | 5.1E-32 |
| *PDCD4* | 1.00759 | 1.07E-33 | 1.3E-31 |
| *KLHL24* | 1.466584 | 2.59E-33 | 3.03E-31 |
| *CNTNAP3* | 1.117295 | 6.19E-33 | 7.07E-31 |
| *SLC7A7* | 1.686685 | 1.95E-32 | 2.17E-30 |
| *TBX3* | 2.5461 | 4.79E-32 | 5.21E-30 |
| *DMKN* | -1.21149 | 8.92E-32 | 9.64E-30 |
| *APOL6* | 1.021415 | 1.54E-31 | 1.63E-29 |
| *MAMDC2* | 1.964506 | 1.96E-31 | 2.05E-29 |
| *SEMA5A* | -1.96149 | 2.78E-31 | 2.89E-29 |
| *SLIT2* | -1.3529 | 3.18E-31 | 3.29E-29 |
| *RPP14* | -1.03149 | 3.31E-31 | 3.4E-29 |
| *STRA6* | 2.090465 | 5.81E-31 | 5.85E-29 |
| *PGAP3* | -1.56953 | 1.88E-30 | 1.87E-28 |
| *DOCK11* | 1.449746 | 2.72E-30 | 2.66E-28 |
| *DLG3* | 1.387303 | 2.9E-30 | 2.8E-28 |
| *BMF* | 1.37521 | 6.42E-30 | 6.08E-28 |
| *NPW* | -1.2721 | 8.43E-30 | 7.9E-28 |
| *ATP9A* | 1.037846 | 1.03E-29 | 9.57E-28 |
| *ALDH1L2* | 1.131601 | 1.63E-29 | 1.48E-27 |
| *WDR45* | 1.009764 | 3.84E-29 | 3.36E-27 |
| *CAMK1D* | 1.445252 | 4.37E-29 | 3.81E-27 |
| *CACNG8* | 2.558938 | 4.53E-29 | 3.92E-27 |
| *GADD45G* | -1.97093 | 7.23E-29 | 6.2E-27 |
| *SLC6A17* | 2.324619 | 7.44E-29 | 6.34E-27 |
| *MPP4* | 2.724983 | 9.52E-29 | 8.03E-27 |
| *TRIM16* | 1.026839 | 3.46E-28 | 2.8E-26 |
| *CFH* | 1.246921 | 6.08E-28 | 4.84E-26 |
| *PKIB* | -1.5164 | 8.72E-28 | 6.79E-26 |
| *C3orf14* | -1.08001 | 1.16E-27 | 8.84E-26 |
| *MAP7* | -1.48928 | 2.61E-27 | 1.92E-25 |
| *CGN* | -1.73468 | 5.34E-27 | 3.87E-25 |
| *ENO3* | -1.1077 | 5.7E-26 | 3.89E-24 |
| *EML1* | 1.333033 | 6.05E-26 | 4.08E-24 |
| *FAM160B1* | 1.086096 | 9.81E-26 | 6.5E-24 |
| *AK3* | 1.113535 | 1.71E-24 | 1.03E-22 |
| *KIAA1456* | 1.601665 | 3.12E-24 | 1.86E-22 |
| *SLC8A1* | -1.75582 | 3.51E-24 | 2.06E-22 |
| *EBI3* | 1.110055 | 6.52E-24 | 3.69E-22 |
| *HMCN1* | -1.6358 | 3.91E-23 | 2.1E-21 |
| *KIAA1211* | 2.086694 | 4.68E-23 | 2.48E-21 |
| *TMEM173* | 3.579916 | 5.28E-23 | 2.76E-21 |
| *PLEKHF1* | -1.55309 | 6.3E-23 | 3.24E-21 |
| *MAP3K7CL* | 1.253402 | 7.03E-23 | 3.61E-21 |
| *CAPN5* | 1.060873 | 9.69E-23 | 4.85E-21 |
| *ARHGEF6* | 1.312344 | 1.13E-22 | 5.58E-21 |
| *DSCAML1* | -1.55958 | 4.37E-22 | 2.05E-20 |
| *CCDC113* | -1.61139 | 1.24E-21 | 5.74E-20 |
| *ADGRF4* | 2.289632 | 2.88E-21 | 1.28E-19 |
| *ZNF585B* | -2.55006 | 3.96E-21 | 1.73E-19 |
| *CCL5* | 1.343492 | 1.67E-20 | 6.99E-19 |
| *EPHA3* | -1.20401 | 3.64E-20 | 1.48E-18 |
| *CYFIP2* | -1.27232 | 5.96E-20 | 2.39E-18 |
| *MYO1D* | -1.00777 | 6.56E-20 | 2.6E-18 |
| *TP73* | 3.866642 | 6.62E-20 | 2.62E-18 |
| *CD82* | 1.208769 | 8.09E-20 | 3.14E-18 |
| *HOOK1* | -1.2775 | 8.98E-20 | 3.47E-18 |
| *JAZF1* | 1.023022 | 1.04E-19 | 3.96E-18 |
| *KCNK2* | -1.93982 | 1.19E-19 | 4.52E-18 |
| *HECA* | 1.04153 | 1.2E-19 | 4.53E-18 |
| *MAF* | 1.660354 | 1.46E-19 | 5.44E-18 |
| *GXYLT2* | -1.19477 | 1.56E-19 | 5.82E-18 |
| *PC* | -1.06434 | 1.73E-19 | 6.42E-18 |
| *MAB21L3* | 2.397469 | 3.13E-19 | 1.11E-17 |
| *PSMC3IP* | -1.05585 | 4.1E-19 | 1.45E-17 |
| *CDYL2* | 1.874285 | 4.51E-19 | 1.58E-17 |
| *SDK1* | 1.469505 | 9.21E-19 | 3.12E-17 |
| *NETO1* | 2.5609 | 1.05E-18 | 3.54E-17 |
| *PLAT* | 1.271654 | 1.55E-18 | 5.15E-17 |
| *MME* | -1.06409 | 2.61E-18 | 8.51E-17 |
| *DBNDD2* | -1.10702 | 4.63E-18 | 1.47E-16 |
| *BTD* | 1.05581 | 6.96E-18 | 2.19E-16 |
| *WDR78* | -1.28092 | 2.08E-17 | 6.28E-16 |
| *KLF9* | 1.783176 | 2.11E-17 | 6.34E-16 |
| *SULT1A1* | 1.035003 | 2.13E-17 | 6.39E-16 |
| *KYNU* | 3.725261 | 2.37E-17 | 7.05E-16 |
| *ANKRD18A* | 1.284945 | 2.68E-17 | 7.92E-16 |
| *ADGRL3* | -3.11195 | 3.28E-17 | 9.57E-16 |
| *PANX2* | 1.332861 | 4.42E-17 | 1.28E-15 |
| *TMEM52B* | 4.319952 | 7.96E-17 | 2.25E-15 |
| *CPA4* | 2.861281 | 8.44E-17 | 2.37E-15 |
| *COLEC12* | -2.55865 | 8.82E-17 | 2.48E-15 |
| *PCMTD1* | 1.254534 | 1.8E-16 | 4.95E-15 |
| *BAHCC1* | 1.179304 | 2.68E-16 | 7.19E-15 |
| *ARSE* | 1.111308 | 2.9E-16 | 7.73E-15 |
| *C1orf50* | -1.25311 | 3E-16 | 7.96E-15 |
| *RYR2* | 1.388006 | 3.04E-16 | 8.06E-15 |
| *GPR173* | 1.05388 | 3.53E-16 | 9.3E-15 |
| *TFAP2C* | 2.731854 | 6.07E-16 | 1.57E-14 |
| *KRT34* | 2.316469 | 6.83E-16 | 1.75E-14 |
| *ROS1* | 1.262952 | 7.84E-16 | 2E-14 |
| *MVB12B* | 1.249932 | 9.24E-16 | 2.33E-14 |
| *RBP7* | -1.33462 | 1.92E-15 | 4.71E-14 |
| *GPRC5C* | 1.176772 | 2.21E-15 | 5.38E-14 |
| *ITGA4* | -7.72754 | 2.88E-15 | 6.94E-14 |
| *JDP2* | 1.333609 | 2.99E-15 | 7.17E-14 |
| *TMEM63C* | -1.87997 | 3.21E-15 | 7.65E-14 |
| *CLIP4* | 1.367634 | 3.4E-15 | 8.05E-14 |
| *NAT8L* | 1.212566 | 4.16E-15 | 9.75E-14 |
| *KCNJ15* | -1.20732 | 6.18E-15 | 1.41E-13 |
| *DTX4* | 1.899206 | 1.4E-14 | 3.09E-13 |
| *EXPH5* | 1.101517 | 1.97E-14 | 4.23E-13 |
| *SPTLC3* | -1.06859 | 2.01E-14 | 4.32E-13 |
| *DPYSL4* | 1.352257 | 2.03E-14 | 4.35E-13 |
| *SLCO2B1* | 2.17474 | 3.11E-14 | 6.55E-13 |
| *SAMD9L* | 1.088601 | 3.43E-14 | 7.17E-13 |
| *FREM2* | -1.65756 | 4.58E-14 | 9.45E-13 |
| *TCP11L2* | 1.495861 | 5.19E-14 | 1.06E-12 |
| *TMEM38A* | -1.33311 | 6.14E-14 | 1.24E-12 |
| *RBM24* | 3.385725 | 9.39E-14 | 1.84E-12 |
| *RGAG1* | 1.834302 | 9.56E-14 | 1.88E-12 |
| *C3orf80* | -1.20839 | 1.08E-13 | 2.11E-12 |
| *TSHZ3* | 1.23826 | 1.59E-13 | 3.02E-12 |
| *NTNG2* | 1.793567 | 1.67E-13 | 3.16E-12 |
| *CYP26B1* | 1.699885 | 2.6E-13 | 4.81E-12 |
| *TRHDE* | 1.989071 | 3.27E-13 | 6E-12 |
| *SOCS2* | 1.452558 | 3.4E-13 | 6.21E-12 |
| *LPIN3* | 1.01236 | 3.8E-13 | 6.89E-12 |
| *PRSS8* | -1.01346 | 4.37E-13 | 7.85E-12 |
| *SMAD6* | 1.185284 | 4.4E-13 | 7.89E-12 |
| *MAPK8IP2* | -1.03577 | 5.2E-13 | 9.2E-12 |
| *TLE2* | 1.590833 | 6.4E-13 | 1.13E-11 |
| *NOG* | 1.142769 | 6.53E-13 | 1.15E-11 |
| *NDRG4* | 1.014743 | 7.58E-13 | 1.32E-11 |
| *GBP4* | 2.392591 | 8.86E-13 | 1.53E-11 |
| *NIF3L1* | -1.59674 | 1.13E-12 | 1.92E-11 |
| *CAMK1* | -1.03484 | 1.27E-12 | 2.16E-11 |
| *CREBRF* | 1.307221 | 1.44E-12 | 2.43E-11 |
| *RAP1GAP2* | 1.418659 | 1.48E-12 | 2.49E-11 |
| *LRRC15* | 1.620901 | 1.85E-12 | 3.05E-11 |
| *UGT2B4* | 4.260459 | 1.86E-12 | 3.06E-11 |
| *SRSF12* | -1.70033 | 1.91E-12 | 3.15E-11 |
| *GCNT3* | 1.045636 | 1.98E-12 | 3.25E-11 |
| *ARMC3* | -7.30623 | 2.43E-12 | 3.93E-11 |
| *PRSS35* | 1.727188 | 2.8E-12 | 4.49E-11 |
| *PABPC4L* | 1.068259 | 3.04E-12 | 4.85E-11 |
| *KIAA1324L* | 1.03144 | 4.17E-12 | 6.52E-11 |
| *SCD5* | -1.06357 | 4.5E-12 | 6.98E-11 |
| *CYP27A1* | 1.395975 | 4.75E-12 | 7.34E-11 |
| *PGGHG* | 1.030793 | 5.07E-12 | 7.78E-11 |
| *FBLIM1* | -1.7459 | 5.57E-12 | 8.47E-11 |
| *NEFL* | -1.24954 | 5.8E-12 | 8.77E-11 |
| *CSPG4* | 1.588991 | 8.78E-12 | 1.3E-10 |
| *HAVCR2* | 2.309633 | 1.02E-11 | 1.5E-10 |
| *VEPH1* | -2.24903 | 1.17E-11 | 1.7E-10 |
| *BMPER* | 1.128477 | 1.33E-11 | 1.93E-10 |
| *EVI2A* | 1.555996 | 1.4E-11 | 2.02E-10 |
| *FOXO4* | 1.094087 | 1.44E-11 | 2.08E-10 |
| *ANO5* | 1.469456 | 2E-11 | 2.82E-10 |
| *FPR1* | 2.45239 | 2.47E-11 | 3.46E-10 |
| *TMOD2* | 1.383769 | 2.94E-11 | 4.1E-10 |
| *ENPEP* | -1.209 | 3.44E-11 | 4.75E-10 |
| *HMBOX1* | 1.118406 | 3.54E-11 | 4.87E-10 |
| *TLR3* | 1.458301 | 3.94E-11 | 5.37E-10 |
| *PAX2* | 1.671379 | 4.04E-11 | 5.5E-10 |
| *C3AR1* | 1.759861 | 4.64E-11 | 6.26E-10 |
| *TRIML2* | 4.781922 | 5.06E-11 | 6.74E-10 |
| *RBM20* | 1.184414 | 5.22E-11 | 6.95E-10 |
| *ABCC2* | 1.107954 | 5.28E-11 | 7.02E-10 |
| *JAK2* | 1.035904 | 6.21E-11 | 8.15E-10 |
| *DHRS2* | 1.286575 | 1.01E-10 | 1.28E-09 |
| *FAM214A* | 1.041858 | 1.19E-10 | 1.5E-09 |
| *S100A3* | -1.01389 | 1.22E-10 | 1.54E-09 |
| *L3MBTL4* | -2.15439 | 1.27E-10 | 1.59E-09 |
| *RBM43* | 1.622126 | 1.45E-10 | 1.81E-09 |
| *TINAG* | -1.29342 | 1.55E-10 | 1.92E-09 |
| *RNF223* | -2.49543 | 1.56E-10 | 1.93E-09 |
| *SLC22A3* | 1.097679 | 1.8E-10 | 2.21E-09 |
| *AASS* | 1.067804 | 1.89E-10 | 2.31E-09 |
| *ATP8B3* | 1.125237 | 2.17E-10 | 2.64E-09 |
| *GSTT1* | -1.08943 | 2.55E-10 | 3.07E-09 |
| *ULBP1* | 1.197692 | 2.97E-10 | 3.54E-09 |
| *SLIT3* | 1.315718 | 3.29E-10 | 3.91E-09 |
| *CREG2* | -1.73929 | 3.39E-10 | 4.02E-09 |
| *CAMK1G* | -3.29429 | 3.41E-10 | 4.03E-09 |
| *HCN4* | 2.335646 | 4.43E-10 | 5.15E-09 |
| *HR* | 1.009468 | 4.8E-10 | 5.55E-09 |
| *DNER* | 2.235455 | 5.12E-10 | 5.86E-09 |
| *MKX* | -1.73328 | 5.78E-10 | 6.56E-09 |
| *MYPN* | 2.112792 | 1.31E-09 | 1.41E-08 |
| *PLPPR3* | 2.92678 | 1.33E-09 | 1.44E-08 |
| *LEF1* | 2.174713 | 1.36E-09 | 1.46E-08 |
| *HES7* | -1.52542 | 1.53E-09 | 1.63E-08 |
| *TNFRSF18* | -1.31187 | 1.71E-09 | 1.82E-08 |
| *CYP4F11* | 1.930582 | 1.83E-09 | 1.92E-08 |
| *CHN1* | 1.199588 | 1.89E-09 | 1.99E-08 |
| *SHROOM2* | 1.072084 | 2.32E-09 | 2.4E-08 |
| *FAT3* | 3.334861 | 2.66E-09 | 2.72E-08 |
| *PPFIA4* | 1.17282 | 2.76E-09 | 2.82E-08 |
| *P2RY6* | 1.431608 | 4.41E-09 | 4.34E-08 |
| *NEBL* | 1.532153 | 4.61E-09 | 4.52E-08 |
| *PITX1* | 1.004163 | 4.64E-09 | 4.54E-08 |
| *GAREM1* | 1.00444 | 4.74E-09 | 4.64E-08 |
| *LXN* | -4.11651 | 4.81E-09 | 4.7E-08 |
| *GDF7* | 1.383573 | 4.94E-09 | 4.81E-08 |
| *PITPNC1* | 1.082371 | 5.58E-09 | 5.4E-08 |
| *MMP10* | 2.253001 | 7.05E-09 | 6.71E-08 |
| *COL5A1* | 1.489341 | 7.08E-09 | 6.73E-08 |
| *RASD2* | 1.093639 | 7.85E-09 | 7.41E-08 |
| *WDR97* | 1.682761 | 8.33E-09 | 7.81E-08 |
| *CDRT1* | 1.46917 | 8.59E-09 | 8.03E-08 |
| *SPON1* | 1.219903 | 1.16E-08 | 1.06E-07 |
| *NPAS2* | 1.091617 | 1.19E-08 | 1.09E-07 |
| *SYCE2* | -1.02793 | 1.35E-08 | 1.23E-07 |
| *ADAMTS2* | 7.041655 | 1.48E-08 | 1.34E-07 |
| *PRDM8* | 1.72283 | 1.55E-08 | 1.4E-07 |
| *FOXS1* | 1.239315 | 1.68E-08 | 1.51E-07 |
| *PDE4D* | 1.11128 | 1.73E-08 | 1.55E-07 |
| *LAPTM5* | 2.422483 | 1.88E-08 | 1.68E-07 |
| *TNFAIP6* | 1.835034 | 1.89E-08 | 1.68E-07 |
| *NIPAL2* | 1.178791 | 1.98E-08 | 1.76E-07 |
| *GAL3ST4* | 1.109635 | 2.03E-08 | 1.8E-07 |
| *AMTN* | 3.092319 | 2.12E-08 | 1.88E-07 |
| *GABBR1* | 1.080071 | 2.13E-08 | 1.88E-07 |
| *CDH18* | -3.80317 | 2.46E-08 | 2.16E-07 |
| *PABPC1L* | 1.251211 | 2.68E-08 | 2.34E-07 |
| *PIR* | 1.064994 | 2.7E-08 | 2.35E-07 |
| *GRIK2* | -1.03158 | 2.88E-08 | 2.49E-07 |
| *GIPC3* | -2.16949 | 2.95E-08 | 2.54E-07 |
| *FLRT3* | 1.306067 | 3.11E-08 | 2.67E-07 |
| *MBOAT1* | 1.047628 | 3.28E-08 | 2.81E-07 |
| *NEFM* | -1.36104 | 3.39E-08 | 2.89E-07 |
| *HSD17B8* | -1.03771 | 3.4E-08 | 2.9E-07 |
| *SLC4A11* | 1.03993 | 3.97E-08 | 3.36E-07 |
| *CXCL11* | 3.863287 | 4.14E-08 | 3.5E-07 |
| *SPTB* | 1.067359 | 4.18E-08 | 3.52E-07 |
| *GLI1* | 1.327121 | 4.27E-08 | 3.59E-07 |
| *KRT15* | 2.271532 | 4.46E-08 | 3.74E-07 |
| *FOXL2NB* | 4.333966 | 4.87E-08 | 4.05E-07 |
| *USP35* | 1.032921 | 4.93E-08 | 4.09E-07 |
| *HPN* | -2.04523 | 5.05E-08 | 4.18E-07 |
| *MFNG* | 1.452417 | 5.77E-08 | 4.74E-07 |
| *CORO2B* | 4.082622 | 6.05E-08 | 4.94E-07 |
| *SLCO1B3* | 6.820809 | 6.61E-08 | 5.35E-07 |
| *ZNF334* | -5.03898 | 7.04E-08 | 5.68E-07 |
| *PRR16* | 1.195176 | 1.02E-07 | 7.97E-07 |
| *IL21R* | 1.541714 | 1.02E-07 | 8E-07 |
| *AQP1* | 2.191588 | 1.16E-07 | 8.97E-07 |
| *FLRT1* | 2.859033 | 1.17E-07 | 9.05E-07 |
| *SLC1A3* | -1.06108 | 1.19E-07 | 9.18E-07 |
| *LAMP3* | -2.18464 | 1.22E-07 | 9.43E-07 |
| *SLC29A3* | 1.279591 | 1.23E-07 | 9.45E-07 |
| *FAHD2B* | -1.28797 | 1.27E-07 | 9.74E-07 |
| *SDR42E1* | -5.27595 | 1.31E-07 | 9.99E-07 |
| *ZNF704* | 2.445214 | 1.35E-07 | 1.03E-06 |
| *FAM83F* | -2.92746 | 1.8E-07 | 1.35E-06 |
| *ICK* | 1.167691 | 1.82E-07 | 1.36E-06 |
| *DCLK2* | 1.165174 | 1.87E-07 | 1.39E-06 |
| *KCNJ16* | 1.741073 | 1.94E-07 | 1.44E-06 |
| *AKR1C1* | 2.352543 | 1.97E-07 | 1.46E-06 |
| *NPR3* | -2.854 | 2.29E-07 | 1.67E-06 |
| *ZNF469* | 1.393527 | 2.45E-07 | 1.79E-06 |
| *C3orf18* | 1.857765 | 2.72E-07 | 1.97E-06 |
| *ARC* | -1.35419 | 2.79E-07 | 2.02E-06 |
| *PCDHGA7* | -1.68062 | 3.26E-07 | 2.33E-06 |
| *OLAH* | 1.943937 | 3.41E-07 | 2.42E-06 |
| *APOBEC3G* | 1.822268 | 3.43E-07 | 2.44E-06 |
| *ARHGAP6* | -1.19083 | 4.58E-07 | 3.19E-06 |
| *ALX4* | 2.871858 | 4.85E-07 | 3.36E-06 |
| *CCL2* | 2.25799 | 4.89E-07 | 3.39E-06 |
| *GRID1* | 1.328006 | 5.05E-07 | 3.49E-06 |
| *FAM188B* | 1.808942 | 5.16E-07 | 3.55E-06 |
| *SULT1A2* | 2.052674 | 5.37E-07 | 3.69E-06 |
| *ADAMTS10* | 1.240973 | 5.5E-07 | 3.77E-06 |
| *GLIS1* | 2.221551 | 5.54E-07 | 3.8E-06 |
| *PALD1* | 2.637791 | 5.73E-07 | 3.92E-06 |
| *TBC1D7* | 1.226298 | 5.87E-07 | 4E-06 |
| *GSTA4* | 1.37132 | 5.94E-07 | 4.05E-06 |
| *FBLN5* | 2.192369 | 5.97E-07 | 4.07E-06 |
| *DOCK3* | 1.352885 | 7.09E-07 | 4.77E-06 |
| *TAL1* | -2.72612 | 7.29E-07 | 4.9E-06 |
| *VSIR* | 1.407364 | 7.31E-07 | 4.91E-06 |
| *TNFSF15* | -1.45306 | 7.56E-07 | 5.06E-06 |
| *XDH* | 1.40629 | 7.76E-07 | 5.19E-06 |
| *SPNS3* | -1.15934 | 8.67E-07 | 5.76E-06 |
| *EFHB* | 1.104336 | 9.52E-07 | 6.27E-06 |
| *SLC16A8* | 1.82158 | 1.08E-06 | 7.09E-06 |
| *RBP5* | 1.064213 | 1.09E-06 | 7.13E-06 |
| *ANGPT4* | 1.757318 | 1.16E-06 | 7.55E-06 |
| *CCDC154* | 1.281162 | 1.18E-06 | 7.66E-06 |
| *FMN2* | 4.264234 | 1.2E-06 | 7.75E-06 |
| *LRP1B* | 1.352035 | 1.22E-06 | 7.89E-06 |
| *LOC101929372* | 1.640741 | 1.33E-06 | 8.53E-06 |
| *LRP5L* | 1.230832 | 1.35E-06 | 8.7E-06 |
| *ACP7* | 1.102234 | 1.45E-06 | 9.24E-06 |
| *FOXO6* | -1.33218 | 1.47E-06 | 9.37E-06 |
| *TMEFF2* | 6.209067 | 1.49E-06 | 9.49E-06 |
| *RASSF10* | -1.00223 | 1.71E-06 | 1.08E-05 |
| *PRH1* | -1.4407 | 1.76E-06 | 1.11E-05 |
| *POSTN* | 1.493174 | 1.78E-06 | 1.11E-05 |
| *SLC19A3* | -6.51431 | 1.83E-06 | 1.15E-05 |
| *VIP* | 1.185006 | 2.02E-06 | 1.26E-05 |
| *SPACA6* | 1.051156 | 2.04E-06 | 1.26E-05 |
| *EGFL8* | 1.077982 | 2.43E-06 | 1.49E-05 |
| *FOXL2* | 2.909456 | 2.93E-06 | 1.76E-05 |
| *AMBP* | 1.983337 | 2.95E-06 | 1.77E-05 |
| *COL4A4* | 1.537168 | 3.07E-06 | 1.83E-05 |
| *EID3* | 1.557117 | 4.05E-06 | 2.36E-05 |
| *ID4* | 3.93806 | 4.35E-06 | 2.53E-05 |
| *ZNF662* | 1.235918 | 4.44E-06 | 2.57E-05 |
| *GPR146* | 1.0533 | 4.51E-06 | 2.61E-05 |
| *C7* | 6.291128 | 4.54E-06 | 2.63E-05 |
| *KRTAP5-2* | -1.30192 | 4.57E-06 | 2.64E-05 |
| *ETV7* | 1.465661 | 4.71E-06 | 2.72E-05 |
| *TBX2* | 1.234281 | 4.72E-06 | 2.72E-05 |
| *IGSF10* | 6.16007 | 5.79E-06 | 3.28E-05 |
| *EML6* | -1.28164 | 5.96E-06 | 3.37E-05 |
| *NUTM2B* | -4.15908 | 6.39E-06 | 3.6E-05 |
| *DCHS2* | -1.34797 | 6.67E-06 | 3.74E-05 |
| *BBS5* | -1.02589 | 6.79E-06 | 3.8E-05 |
| *RASGRF2* | -1.15731 | 6.89E-06 | 3.85E-05 |
| *GRIP2* | 1.262942 | 7.24E-06 | 4.03E-05 |
| *KLHL4* | 6.141845 | 7.76E-06 | 4.29E-05 |
| *DLX3* | 1.557373 | 7.84E-06 | 4.34E-05 |
| *FNDC5* | -1.75413 | 8.86E-06 | 4.86E-05 |
| *CCNB3* | 1.421271 | 1.03E-05 | 5.6E-05 |
| *TTYH2* | 1.207097 | 1.03E-05 | 5.61E-05 |
| *RENBP* | -2.1769 | 1.06E-05 | 5.72E-05 |
| *HCLS1* | 2.050884 | 1.16E-05 | 6.26E-05 |
| *SLC25A18* | 1.040785 | 1.21E-05 | 6.51E-05 |
| *SEMA3D* | 1.761585 | 1.23E-05 | 6.57E-05 |
| *IL34* | -5.98223 | 1.27E-05 | 6.76E-05 |
| *FLVCR2* | 1.41262 | 1.28E-05 | 6.8E-05 |
| *MSS51* | 1.239542 | 1.28E-05 | 6.82E-05 |
| *ANOS1* | 1.898056 | 1.34E-05 | 7.1E-05 |
| *SLC25A27* | 3.170219 | 1.37E-05 | 7.26E-05 |
| *PSG3* | -1.87316 | 1.44E-05 | 7.6E-05 |
| *COL3A1* | -1.23002 | 1.53E-05 | 8.02E-05 |
| *NPPB* | -2.10798 | 1.56E-05 | 8.18E-05 |
| *SLCO4C1* | -1.12937 | 1.6E-05 | 8.39E-05 |
| *ADRA1D* | 5.886533 | 1.71E-05 | 8.9E-05 |
| *ZNF502* | 1.123387 | 1.72E-05 | 8.94E-05 |
| *BTNL9* | 1.33464 | 1.78E-05 | 9.23E-05 |
| *MAATS1* | -1.31832 | 1.79E-05 | 9.28E-05 |
| *FUT10* | 1.13228 | 1.85E-05 | 9.56E-05 |
| *B3GAT1* | -1.63716 | 1.99E-05 | 0.000102 |
| *ZFYVE28* | 1.230396 | 2.06E-05 | 0.000106 |
| *CARF* | 1.057138 | 2.32E-05 | 0.000118 |
| *CSMD3* | 3.322329 | 2.34E-05 | 0.000119 |
| *PLA2G6* | 1.072365 | 2.37E-05 | 0.00012 |
| *TTC6* | 1.194361 | 2.47E-05 | 0.000125 |
| *NCCRP1* | 1.938839 | 2.59E-05 | 0.00013 |
| *HAS2* | 1.432272 | 2.63E-05 | 0.000132 |
| *VNN2* | 2.483504 | 2.84E-05 | 0.000141 |
| *LOC100130451* | 5.949735 | 2.87E-05 | 0.000143 |
| *TMCO5A* | -5.96842 | 2.96E-05 | 0.000147 |
| *PINLYP* | 1.024447 | 3.11E-05 | 0.000154 |
| *OVOL2* | -4.37376 | 3.14E-05 | 0.000155 |
| *ZNF391* | -1.59187 | 3.21E-05 | 0.000158 |
| *CKMT1B* | -6.09473 | 3.49E-05 | 0.00017 |
| *ERC2* | 1.151902 | 3.54E-05 | 0.000172 |
| *FBXW10* | 1.642075 | 3.76E-05 | 0.000181 |
| *ZBTB3* | 1.226793 | 3.98E-05 | 0.000191 |
| *MAP1A* | 1.220216 | 4.24E-05 | 0.000203 |
| *GABRB1* | -5.92804 | 4.35E-05 | 0.000207 |
| *RGPD1* | 2.943518 | 4.59E-05 | 0.000218 |
| *CCDC78* | 1.00857 | 4.59E-05 | 0.000218 |
| *KCNV2* | -1.41305 | 4.61E-05 | 0.000218 |
| *L3MBTL1* | 1.052483 | 4.63E-05 | 0.000219 |
| *KLK10* | 6.011772 | 4.76E-05 | 0.000225 |
| *LCN2* | -1.55484 | 4.78E-05 | 0.000225 |
| *AXIN2* | 1.296284 | 4.94E-05 | 0.000232 |
| *DAAM2* | -1.16985 | 5.07E-05 | 0.000238 |
| *TAC3* | 5.835257 | 5.23E-05 | 0.000245 |
| *HHIPL2* | 1.308879 | 5.35E-05 | 0.00025 |
| *FRAT1* | 1.576181 | 5.54E-05 | 0.000259 |
| *CYGB* | 6.071357 | 6.11E-05 | 0.000282 |
| *IFNL2* | 3.659338 | 6.22E-05 | 0.000287 |
| *SCN9A* | -1.08312 | 6.45E-05 | 0.000296 |
| *TEX19* | 1.667577 | 6.54E-05 | 0.0003 |
| *ROBO4* | 1.289008 | 6.81E-05 | 0.000311 |
| *PLCD1* | 1.515326 | 6.88E-05 | 0.000314 |
| *CCDC88B* | 1.242161 | 6.96E-05 | 0.000317 |
| *LRTM2* | -1.49604 | 7.09E-05 | 0.000323 |
| *RHEBL1* | 1.24832 | 7.25E-05 | 0.00033 |
| *FBXO15* | 3.228092 | 7.76E-05 | 0.00035 |
| *CCSER1* | -2.1793 | 8.64E-05 | 0.000386 |
| *ACTL8* | -2.32414 | 8.68E-05 | 0.000387 |
| *GPR1* | 3.650488 | 8.98E-05 | 0.000399 |
| *SLC3A1* | 2.527539 | 9.02E-05 | 0.000401 |
| *KCNG1* | -1.35385 | 9.28E-05 | 0.000411 |
| *MINOS1-NBL1* | -1.57769 | 9.44E-05 | 0.000418 |
| *CACNA2D3* | 1.06021 | 0.000103 | 0.000451 |
| *ADGRE1* | 5.826675 | 0.000113 | 0.000492 |
| *SLC25A45* | 1.243481 | 0.000117 | 0.000508 |
| *ASB9* | 1.047143 | 0.000117 | 0.000508 |
| *ANXA8* | 2.450923 | 0.00012 | 0.000519 |
| *COX6B2* | -1.14311 | 0.000129 | 0.000551 |
| *PDE4C* | 1.923996 | 0.00013 | 0.000556 |
| *IL10RA* | 1.016227 | 0.000136 | 0.000581 |
| *FBXL7* | 1.528474 | 0.000139 | 0.000591 |
| *ANKRD63* | 5.632144 | 0.000142 | 0.000604 |
| *GJB5* | -2.99774 | 0.000144 | 0.000612 |
| *MYO7A* | 2.83125 | 0.000152 | 0.000644 |
| *GABRR2* | 1.713819 | 0.000157 | 0.000662 |
| *DLL4* | -1.34016 | 0.000162 | 0.000682 |
| *GPR17* | 1.275282 | 0.000168 | 0.000706 |
| *DLX4* | 2.467449 | 0.000171 | 0.000714 |
| *SV2A* | 1.685497 | 0.000172 | 0.000718 |
| *SLC2A5* | 1.030844 | 0.000173 | 0.000725 |
| *PDZD7* | 1.388582 | 0.000186 | 0.000773 |
| *YPEL2* | 1.033393 | 0.000192 | 0.000794 |
| *CAPN3* | 2.023493 | 0.000193 | 0.000796 |
| *COX6A2* | -1.47247 | 0.000194 | 0.0008 |
| *CRHBP* | -4.26752 | 0.000197 | 0.000811 |
| *PTAFR* | -1.1832 | 0.000199 | 0.000819 |
| *PLSCR4* | 1.098682 | 0.000215 | 0.000878 |
| *KIF12* | 1.062614 | 0.000232 | 0.00094 |
| *DPF3* | 1.971839 | 0.000237 | 0.000962 |
| *SLC23A1* | 2.785358 | 0.00024 | 0.000971 |
| *HYKK* | 1.40213 | 0.000244 | 0.000986 |
| *KRT16* | 5.746056 | 0.000248 | 0.000999 |
| *IL18R1* | 1.205104 | 0.000257 | 0.001033 |
| *GPR89B* | -1.1125 | 0.000258 | 0.001038 |
| *TMC5* | 2.53011 | 0.000262 | 0.001053 |
| *GHR* | 1.539436 | 0.000268 | 0.001072 |
| *BCAN* | -1.01408 | 0.000272 | 0.001089 |
| *ACVRL1* | 2.272353 | 0.000275 | 0.001096 |
| *NPHS1* | 2.601302 | 0.000276 | 0.001101 |
| *NEK10* | 1.331371 | 0.000276 | 0.001102 |
| *RANBP3L* | 4.141365 | 0.000285 | 0.001134 |
| *LMNTD2* | 1.174256 | 0.000291 | 0.001153 |
| *CALCR* | 5.483918 | 0.000312 | 0.001228 |
| *PIPOX* | -2.96679 | 0.000326 | 0.001277 |
| *EFR3B* | 1.480619 | 0.000329 | 0.001287 |
| *RASSF6* | -1.17109 | 0.000338 | 0.001317 |
| *PCDHB7* | 5.97557 | 0.000341 | 0.001326 |
| *FHIT* | -1.34923 | 0.000342 | 0.001332 |
| *ATP8A1* | 1.946801 | 0.000344 | 0.001338 |
| *CREB3L3* | 6.02329 | 0.000348 | 0.001352 |
| *CHST6* | 6.047486 | 0.000348 | 0.001352 |
| *DCST1* | -1.0563 | 0.000355 | 0.001377 |
| *DIRAS1* | -1.60491 | 0.000357 | 0.001381 |
| *LRIT3* | 1.396745 | 0.000357 | 0.001381 |
| *UBE2Q2L* | 1.947139 | 0.000359 | 0.001389 |
| *ESR1* | 1.445358 | 0.000368 | 0.001423 |
| *PSORS1C2* | 1.459067 | 0.000384 | 0.001482 |
| *NPR2* | 1.062053 | 0.000386 | 0.001488 |
| *NEURL1* | 1.35392 | 0.000387 | 0.001491 |
| *VCAM1* | -5.50599 | 0.000393 | 0.001509 |
| *ADGRF2* | 1.286027 | 0.0004 | 0.001533 |
| *AVPR2* | 1.181053 | 0.000407 | 0.001561 |
| *EVI2B* | 1.756125 | 0.000411 | 0.001572 |
| *HLA-DMA* | 1.530722 | 0.00042 | 0.001604 |
| *FGF1* | 1.803332 | 0.000422 | 0.001609 |
| *C6orf229* | -2.74869 | 0.000424 | 0.001617 |
| *IFNB1* | 1.09578 | 0.000426 | 0.001622 |
| *NAT2* | 5.47535 | 0.000427 | 0.001627 |
| *IRF6* | -3.26075 | 0.00044 | 0.001668 |
| *ATP6V0D2* | 1.505564 | 0.000446 | 0.001691 |
| *ITGA11* | 5.614663 | 0.000455 | 0.001718 |
| *PGA5* | 3.150715 | 0.000471 | 0.00177 |
| *SLC7A14* | -1.88038 | 0.000476 | 0.001786 |
| *LIPE* | 1.066736 | 0.000481 | 0.001803 |
| *PTGS2* | -1.64182 | 0.000488 | 0.001826 |
| *MEFV* | 1.005031 | 0.000503 | 0.001877 |
| *CD8A* | 2.781147 | 0.000507 | 0.001886 |
| *ACAN* | 2.08278 | 0.00053 | 0.001963 |
| *VEGFD* | 5.724561 | 0.000545 | 0.002012 |
| *XAF1* | 1.325243 | 0.000568 | 0.00209 |
| *ZNF852* | 1.053107 | 0.00059 | 0.002163 |
| *KIAA1324* | 1.055455 | 0.000599 | 0.002194 |
| *FZD3* | 2.629094 | 0.000607 | 0.002221 |
| *CYP2C8* | 5.392578 | 0.000611 | 0.002233 |
| *ELSPBP1* | 3.871893 | 0.000662 | 0.0024 |
| *REEP2* | -1.19128 | 0.000683 | 0.002473 |
| *SFRP4* | -1.56628 | 0.000705 | 0.002543 |
| *PROM2* | 1.147134 | 0.000706 | 0.002545 |
| *RIPPLY3* | -5.58607 | 0.00074 | 0.002657 |
| *LHFPL3* | -3.51542 | 0.000764 | 0.002736 |
| *TMEM86A* | 1.564334 | 0.000771 | 0.002758 |
| *CUBN* | -1.83507 | 0.000772 | 0.002761 |
| *SCGB3A1* | -5.52084 | 0.000787 | 0.002812 |
| *DIO2* | 1.691257 | 0.000797 | 0.002845 |
| *SYTL5* | 1.279147 | 0.000799 | 0.002849 |
| *ADAMTS8* | -5.60281 | 0.000833 | 0.002963 |
| *TBX4* | 5.724631 | 0.000833 | 0.002964 |
| *CARD14* | 1.315965 | 0.00084 | 0.002984 |
| *BMP6* | 1.964225 | 0.000841 | 0.002986 |
| *SLFN12L* | 1.638892 | 0.000846 | 0.003003 |
| *IGLON5* | 2.304871 | 0.000851 | 0.003018 |
| *WNT9A* | 1.367843 | 0.000876 | 0.003096 |
| *KIAA2012* | 2.88765 | 0.000911 | 0.003209 |
| *CLDN3* | -2.30902 | 0.000915 | 0.00322 |
| *SYT3* | 1.028787 | 0.000919 | 0.003234 |
| *CNKSR1* | -1.03336 | 0.000943 | 0.003306 |
| *PTGIS* | -3.04628 | 0.000949 | 0.003321 |
| *SLC25A6* | -1.01128 | 0.000991 | 0.003451 |
| *SNAP25* | 1.029116 | 0.001004 | 0.003491 |
| *ADM2* | -1.85691 | 0.001007 | 0.003499 |
| *BARX2* | -5.74061 | 0.001056 | 0.003653 |
| *LDHAL6A* | 1.890056 | 0.001057 | 0.003655 |
| *ZP4* | -5.41882 | 0.001061 | 0.003667 |
| *ATF7IP2* | 1.113121 | 0.001073 | 0.003702 |
| *SBK1* | -1.04337 | 0.001122 | 0.00386 |
| *SLX1A* | 2.076122 | 0.00115 | 0.003941 |
| *SATB1* | 2.06511 | 0.001211 | 0.004134 |
| *RNF224* | 1.003734 | 0.001217 | 0.004152 |
| *PDE4B* | 5.421166 | 0.00131 | 0.004434 |
| *CALB1* | 3.137291 | 0.001368 | 0.004609 |
| *PSG4* | -2.48664 | 0.001477 | 0.004932 |
| *CD163L1* | 2.002377 | 0.001488 | 0.004964 |
| *IGSF9* | -1.33864 | 0.001548 | 0.005154 |
| *DNAJC15* | 5.126204 | 0.001657 | 0.005477 |
| *ADRA2A* | -1.82808 | 0.00167 | 0.005516 |
| *KRT33B* | 1.775969 | 0.001687 | 0.005567 |
| *CDSN* | 1.048323 | 0.001735 | 0.005715 |
| *CTXN2* | -5.57591 | 0.001738 | 0.005721 |
| *KCNJ10* | 5.4974 | 0.001738 | 0.005721 |
| *PSG8* | -2.22471 | 0.001743 | 0.005733 |
| *LRRC4C* | 3.143716 | 0.001834 | 0.006001 |
| *LRRK2* | 1.704172 | 0.001842 | 0.006025 |
| *ACTRT3* | -2.07955 | 0.001889 | 0.006164 |
| *PRSS22* | -2.20261 | 0.001967 | 0.006395 |
| *IGLL1* | 3.730329 | 0.001981 | 0.006432 |
| *TERB1* | 2.367765 | 0.002025 | 0.006562 |
| *CHAD* | 1.230736 | 0.002036 | 0.00659 |
| *PLCB2* | 3.949965 | 0.002037 | 0.006592 |
| *MYLK4* | 1.047436 | 0.002056 | 0.006646 |
| *HOXC11* | 1.459866 | 0.002207 | 0.00708 |
| *ACTA1* | 1.752294 | 0.002212 | 0.007092 |
| *SCGB2B2* | 1.400743 | 0.002245 | 0.007191 |
| *PEX11G* | 1.245856 | 0.002316 | 0.007391 |
| *B4GALNT2* | 1.319679 | 0.002376 | 0.007552 |
| *LLGL2* | 3.15033 | 0.002436 | 0.007719 |
| *DDIT4L* | 1.541456 | 0.002463 | 0.0078 |
| *ACE2* | -3.4679 | 0.002474 | 0.007832 |
| *C5orf60* | -3.90073 | 0.002494 | 0.007885 |
| *TRIM17* | 2.092145 | 0.002516 | 0.007949 |
| *APOBEC3D* | 5.373683 | 0.002534 | 0.008001 |
| *SESN3* | 1.914909 | 0.002564 | 0.008077 |
| *STXBP5L* | -1.93105 | 0.002618 | 0.008221 |
| *GAS1* | 2.017382 | 0.002638 | 0.008277 |
| *HIST1H2AE* | -1.9933 | 0.00273 | 0.008532 |
| *DENND2C* | 1.015157 | 0.002732 | 0.008537 |
| *KCNRG* | 3.901003 | 0.002787 | 0.008687 |
| *NXPH3* | -1.35805 | 0.002788 | 0.008687 |
| *KCNIP4* | -1.20074 | 0.002884 | 0.008949 |
| *CCDC160* | 5.120319 | 0.002944 | 0.009113 |
| *PRRX2* | -5.42336 | 0.002977 | 0.009192 |
| *PCDHB16* | 5.376166 | 0.002977 | 0.009192 |
| *LIX1* | -5.37342 | 0.002977 | 0.009192 |
| *PIK3C2G* | 5.31366 | 0.002977 | 0.009192 |
| *CEACAM1* | 1.538115 | 0.003084 | 0.009477 |
| *PLXDC1* | 1.648275 | 0.003089 | 0.009489 |
| *KBTBD13* | 2.015804 | 0.003109 | 0.009543 |
| *PLAC1* | 1.272134 | 0.003121 | 0.009575 |
| *CRHR2* | 1.104042 | 0.003136 | 0.009615 |
| *ZNF501* | -1.36454 | 0.003227 | 0.009862 |
| *DRGX* | 2.678769 | 0.003333 | 0.010153 |
| *TP53I11* | -3.15068 | 0.003441 | 0.010457 |
| *MAB21L1* | -5.17084 | 0.003499 | 0.010616 |
| *CSRNP3* | 1.040888 | 0.003537 | 0.010714 |
| *IMPG2* | 1.476619 | 0.003636 | 0.010975 |
| *CLRN3* | -5.06374 | 0.003763 | 0.011311 |
| *SGIP1* | 1.657114 | 0.003786 | 0.01137 |
| *TBR1* | 1.410545 | 0.003834 | 0.011491 |
| *IFNL1* | 1.237029 | 0.003865 | 0.011566 |
| *TMPRSS6* | 1.517583 | 0.003959 | 0.011801 |
| *FIGNL2* | 1.465715 | 0.003966 | 0.011818 |
| *NALCN* | 1.131879 | 0.004028 | 0.011973 |
| *SCN11A* | 2.892348 | 0.004163 | 0.012332 |
| *SULT1A3* | 1.267548 | 0.004174 | 0.01236 |
| *UFSP1* | 1.463643 | 0.004236 | 0.012521 |
| *CHRNB2* | -1.21847 | 0.004254 | 0.012565 |
| *ADAM32* | 1.600893 | 0.004436 | 0.013023 |
| *NPY4R* | 2.132552 | 0.004474 | 0.013119 |
| *MYO16* | 4.91063 | 0.004478 | 0.013126 |
| *KCNJ2* | -1.60816 | 0.004516 | 0.013229 |
| *BOP1* | 2.38004 | 0.004865 | 0.014131 |
| *ZNF835* | 1.192089 | 0.004915 | 0.014264 |
| *ADGRF3* | 1.10851 | 0.004954 | 0.01436 |
| *ZNF521* | 2.479781 | 0.005066 | 0.014652 |
| *IFNA5* | 3.669753 | 0.005109 | 0.014765 |
| *C1QTNF1* | 1.379751 | 0.00518 | 0.01495 |
| *CXCL10* | 2.235191 | 0.005207 | 0.015017 |
| *EPHB3* | 2.401081 | 0.005247 | 0.015112 |
| *PTPRN* | 1.630024 | 0.005304 | 0.015244 |
| *GSC2* | -5.17802 | 0.005335 | 0.015315 |
| *DCDC2C* | 5.125918 | 0.005335 | 0.015315 |
| *CHST9* | -5.20761 | 0.005335 | 0.015315 |
| *DUSP13* | 5.147026 | 0.005335 | 0.015315 |
| *PKDREJ* | -1.59431 | 0.005338 | 0.015322 |
| *METTL7A* | 2.33385 | 0.005342 | 0.01533 |
| *RARB* | 4.901562 | 0.005373 | 0.015411 |
| *CST6* | -1.43276 | 0.005546 | 0.015835 |
| *MYOZ1* | 1.911636 | 0.005683 | 0.01618 |
| *XKR9* | 1.037699 | 0.005857 | 0.016628 |
| *LMO7DN* | 3.595149 | 0.005873 | 0.016662 |
| *LRRC29* | 1.174027 | 0.005879 | 0.016674 |
| *C10orf126* | -1.45767 | 0.005909 | 0.016739 |
| *GABRA5* | 2.856363 | 0.006016 | 0.017008 |
| *TMEM150C* | 2.783073 | 0.006021 | 0.017019 |
| *OIT3* | 2.919134 | 0.006145 | 0.017316 |
| *ALG1L2* | -2.75157 | 0.006235 | 0.017558 |
| *TXNRD3NB* | 3.471525 | 0.006262 | 0.017624 |
| *NAPSA* | -2.59398 | 0.006306 | 0.017732 |
| *SYT8* | 3.499377 | 0.006425 | 0.018039 |
| *RNASE7* | -2.55359 | 0.006441 | 0.018079 |
| *RGS11* | 3.482833 | 0.006505 | 0.018251 |
| *ZNF732* | -1.11267 | 0.006588 | 0.018441 |
| *LGR6* | 1.152562 | 0.006692 | 0.018675 |
| *C9orf24* | 1.752702 | 0.006727 | 0.018761 |
| *HRASLS2* | 4.90736 | 0.006778 | 0.018877 |
| *CDH17* | -2.4479 | 0.006809 | 0.018948 |
| *XYLT1* | 1.091774 | 0.00681 | 0.018948 |
| *MDH1B* | 1.392088 | 0.006928 | 0.01923 |
| *TFCP2L1* | 1.075503 | 0.006994 | 0.019369 |
| *PRR19* | 1.222734 | 0.006997 | 0.019374 |
| *ZKSCAN7* | -2.32383 | 0.007113 | 0.019676 |
| *KCNIP2* | 1.042292 | 0.007155 | 0.01977 |
| *CLDN10* | 3.548687 | 0.007165 | 0.019797 |
| *SYN3* | 1.493821 | 0.00726 | 0.020021 |
| *SEZ6* | 5.079636 | 0.007285 | 0.020072 |
| *CACNA1C* | -5.04298 | 0.007285 | 0.020072 |
| *UBQLNL* | 4.994483 | 0.007285 | 0.020072 |
| *CALHM3* | 5.086281 | 0.007285 | 0.020072 |
| *RGPD4* | 1.005236 | 0.007294 | 0.020089 |
| *GPC5* | -2.00619 | 0.007328 | 0.020166 |
| *ANKRD30B* | 1.181934 | 0.007386 | 0.020297 |
| *ALS2CR12* | 1.212383 | 0.007429 | 0.020395 |
| *ITGB7* | 1.414183 | 0.007436 | 0.020406 |
| *PLEKHH2* | 1.368821 | 0.00754 | 0.020656 |
| *A1CF* | 2.822769 | 0.007837 | 0.021384 |
| *SLC17A7* | 1.985938 | 0.007977 | 0.021718 |
| *CHRNB1* | 1.038827 | 0.008021 | 0.021819 |
| *TMEM191C* | 1.356361 | 0.008221 | 0.022299 |
| *PTK7* | -1.70702 | 0.008296 | 0.022455 |
| *KCND2* | -3.38735 | 0.008362 | 0.022594 |
| *ZFP14* | 1.61042 | 0.008467 | 0.022836 |
| *VTCN1* | 1.829208 | 0.008516 | 0.022948 |
| *SLC22A13* | 2.881649 | 0.008603 | 0.023172 |
| *RRH* | 1.077902 | 0.008614 | 0.023194 |
| *NAT6* | 1.145323 | 0.008647 | 0.023271 |
| *NFIA* | 2.033457 | 0.008883 | 0.023811 |
| *TRIM69* | -1.38217 | 0.009152 | 0.024454 |
| *PDZD3* | 2.325544 | 0.009164 | 0.024478 |
| *ZBTB7C* | -2.43749 | 0.009185 | 0.024518 |
| *LINC00961* | -1.74537 | 0.009226 | 0.024617 |
| *C15orf56* | -3.07926 | 0.00932 | 0.024837 |
| *ARHGEF15* | -4.78022 | 0.009412 | 0.025037 |
| *LY96* | 1.123743 | 0.009555 | 0.025367 |
| *AZIN2* | 1.136602 | 0.009591 | 0.025451 |
| *HTRA4* | 4.933025 | 0.009614 | 0.0255 |
| *BLID* | 3.124311 | 0.00963 | 0.025532 |
| *SLC1A1* | -1.30924 | 0.009657 | 0.025596 |
| *IGSF11* | 2.610206 | 0.009842 | 0.026044 |
| *CYP2W1* | 1.226963 | 0.009934 | 0.026271 |
| *GNRH2* | 1.0497 | 0.009944 | 0.026287 |
| *RPS17* | 1.524354 | 0.009977 | 0.026366 |
| *IFNL3* | 1.630698 | 0.01007 | 0.026581 |
| *ARHGAP36* | -4.92411 | 0.010096 | 0.026581 |
| *ITIH6* | 4.955556 | 0.010096 | 0.026581 |
| *RAI2* | 4.910401 | 0.010096 | 0.026581 |
| *TG* | 4.948057 | 0.010096 | 0.026581 |
| *NOX3* | 4.88125 | 0.010096 | 0.026581 |
| *RBM11* | -4.92411 | 0.010096 | 0.026581 |
| *GATA5* | 4.903129 | 0.010096 | 0.026581 |
| *UNC13C* | 4.940842 | 0.010096 | 0.026581 |
| *MEDAG* | -4.96409 | 0.010096 | 0.026581 |
| *OR52B2* | 4.86661 | 0.010096 | 0.026581 |
| *HSD11B1* | 4.926082 | 0.010096 | 0.026581 |
| *COL15A1* | 1.121953 | 0.010121 | 0.026637 |
| *PARK2* | 1.374322 | 0.010416 | 0.027326 |
| *SLC9A2* | -1.06737 | 0.010559 | 0.02766 |
| *COL5A3* | 1.727958 | 0.010567 | 0.027675 |
| *RTN4RL1* | 2.047793 | 0.010785 | 0.028187 |
| *RASIP1* | -2.2813 | 0.01088 | 0.028399 |
| *CCL20* | -1.23408 | 0.010882 | 0.028399 |
| *RCOR2* | -1.63166 | 0.010929 | 0.028508 |
| *FAM72C* | -1.04274 | 0.010978 | 0.028621 |
| *LIPI* | -1.74416 | 0.010985 | 0.028635 |
| *SLAMF8* | 1.071175 | 0.011017 | 0.028695 |
| *ZNF404* | -1.39418 | 0.011285 | 0.029291 |
| *LYG1* | 1.031029 | 0.011293 | 0.029305 |
| *PCDH12* | 1.156601 | 0.011347 | 0.029422 |
| *KCTD19* | 1.251616 | 0.011539 | 0.029838 |
| *FAM81B* | 4.804268 | 0.011737 | 0.030272 |
| *SLX1B* | -1.05666 | 0.011754 | 0.030307 |
| *PRDM1* | 2.387036 | 0.011791 | 0.030388 |
| *SBK3* | 2.622208 | 0.011999 | 0.030841 |
| *NLRP5* | -1.86739 | 0.012168 | 0.031255 |
| *TRPV2* | -2.63737 | 0.012205 | 0.031329 |
| *CLEC7A* | 2.048966 | 0.012425 | 0.031828 |
| *GOLGA6L9* | 1.166679 | 0.012485 | 0.031967 |
| *MSH4* | 1.064255 | 0.012562 | 0.032129 |
| *POU3F2* | 1.112237 | 0.012721 | 0.032472 |
| *PHOSPHO1* | 1.479671 | 0.012846 | 0.03274 |
| *ELOA2* | -2.48754 | 0.013174 | 0.03346 |
| *CCDC188* | 1.283153 | 0.013182 | 0.033473 |
| *CATSPERD* | -1.66488 | 0.013436 | 0.034045 |
| *DNMT3L* | 1.863714 | 0.013479 | 0.034142 |
| *EFCAB12* | -2.00489 | 0.013513 | 0.034208 |
| *TBX10* | 3.341371 | 0.013683 | 0.034611 |
| *TEX12* | 3.038296 | 0.013749 | 0.034756 |
| *PLAC4* | 4.664109 | 0.0138 | 0.034852 |
| *OR1F1* | 2.666641 | 0.014195 | 0.035694 |
| *CRH* | 4.76646 | 0.014222 | 0.035694 |
| *PGC* | 4.69888 | 0.014222 | 0.035694 |
| *SLC2A2* | -4.77037 | 0.014222 | 0.035694 |
| *GDAP1L1* | 4.78155 | 0.014222 | 0.035694 |
| *TMEM163* | 4.746882 | 0.014222 | 0.035694 |
| *NLRP12* | -4.88781 | 0.014222 | 0.035694 |
| *ABCA6* | 4.773865 | 0.014222 | 0.035694 |
| *TRIM34* | -4.78768 | 0.014222 | 0.035694 |
| *MYBPHL* | 4.789225 | 0.014222 | 0.035694 |
| *ATOH8* | 1.863136 | 0.014462 | 0.03619 |
| *ZNF843* | -1.04657 | 0.014468 | 0.036197 |
| *TLE6* | 1.35768 | 0.014492 | 0.036242 |
| *CLUL1* | -1.87637 | 0.014547 | 0.036344 |
| *CD72* | 1.503797 | 0.014651 | 0.036572 |
| *TNFSF8* | 2.829012 | 0.014932 | 0.03718 |
| *PMEL* | 1.098694 | 0.015213 | 0.037816 |
| *MKRN2OS* | 2.687299 | 0.01582 | 0.039095 |
| *FXYD6* | 1.416908 | 0.015871 | 0.039191 |
| *SFRP5* | 2.531401 | 0.016241 | 0.039981 |
| *BTBD8* | 1.214168 | 0.016264 | 0.040026 |
| *MST1R* | 1.144815 | 0.016429 | 0.040377 |
| *SPDEF* | -1.11448 | 0.01649 | 0.04052 |
| *RSPH6A* | 2.421277 | 0.016883 | 0.041346 |
| *UNC5B* | 1.152253 | 0.016926 | 0.041441 |
| *HIST1H2AK* | 1.125103 | 0.016938 | 0.041458 |
| *SLC7A8* | 1.980191 | 0.017004 | 0.041606 |
| *LIN28A* | -2.19749 | 0.017062 | 0.041723 |
| *UTS2B* | 1.76859 | 0.017093 | 0.041792 |
| *SPOCK3* | 4.672924 | 0.017396 | 0.042418 |
| *RP1L1* | 1.826893 | 0.017449 | 0.042501 |
| *THEG* | 1.475505 | 0.01758 | 0.042762 |
| *PRAP1* | -1.2796 | 0.017653 | 0.042914 |
| *ZNF572* | 1.131268 | 0.017707 | 0.043038 |
| *WNT10A* | -1.13337 | 0.017833 | 0.043325 |
| *C11orf91* | 1.098424 | 0.018034 | 0.043766 |
| *LINGO1* | 2.630727 | 0.018103 | 0.043908 |
| *LGI2* | -1.20635 | 0.018264 | 0.044246 |
| *CHRNA9* | 1.734996 | 0.018514 | 0.04475 |
| *MAGED4* | 1.083137 | 0.018518 | 0.044753 |
| *HIST1H2BF* | 1.631092 | 0.018598 | 0.044912 |
| *GNAT1* | 1.011317 | 0.018643 | 0.045002 |
| *RNF128* | -1.61925 | 0.018673 | 0.045053 |
| *COMP* | -3.10171 | 0.018694 | 0.045096 |
| *TNFRSF10C* | -3.31895 | 0.018792 | 0.045287 |
| *PCP2* | 1.863309 | 0.018942 | 0.045605 |
| *DEC1* | -1.01548 | 0.019738 | 0.047304 |
| *MOB4* | 2.442082 | 0.019845 | 0.047547 |
| *HIST1H2AM* | 1.452023 | 0.019854 | 0.047558 |
| *MCF2* | 4.632811 | 0.0204 | 0.048581 |
| *ENHO* | 4.632521 | 0.0204 | 0.048581 |
| *SH3GL2* | -4.69717 | 0.0204 | 0.048581 |
| *CLVS1* | -4.63591 | 0.0204 | 0.048581 |
| *MOGAT3* | 4.649386 | 0.0204 | 0.048581 |
| *BTN1A1* | 4.6162 | 0.0204 | 0.048581 |
| *ZNF385B* | 4.632521 | 0.0204 | 0.048581 |
| *ACTG2* | -4.63996 | 0.0204 | 0.048581 |
| *FPR3* | 4.640667 | 0.0204 | 0.048581 |
| *HAMP* | 4.648803 | 0.0204 | 0.048581 |
| *SLC14A2* | -4.64613 | 0.0204 | 0.048581 |
| *TNFRSF17* | 4.640667 | 0.0204 | 0.048581 |
| *CIITA* | -4.63591 | 0.0204 | 0.048581 |
| *NYNRIN* | 4.6162 | 0.0204 | 0.048581 |
| *B3GALT2* | -4.61127 | 0.0204 | 0.048581 |
| *KANK4* | -4.61127 | 0.0204 | 0.048581 |
| *CTRC* | -4.61127 | 0.0204 | 0.048581 |
| *LOC150051* | -1.09705 | 0.020536 | 0.04884 |
| *HORMAD1* | 2.271943 | 0.020569 | 0.048903 |
| *SCN4A* | 2.923881 | 0.020674 | 0.049123 |
